# The Climate Hazards Center Infrared Precipitation with Stations, Version 3

**DOI:** 10.1038/s41597-026-07096-4

**Published:** 2026-04-11

**Authors:** Chris Funk, Pete Peterson, Laura Harrison, Robert Saldivar, Martin Landsfeld, Diego Pedreros, Shraddhanand Shukla, Andreas H. Fink, Frank Davenport, Seth Peterson, William Turner, Austin Sonnier, Michael Budde, Karyn Tabor, James Verdin, Disha Hauzaree, Mohamed Naim, Daniella Alaso, Gregory Husak

**Affiliations:** 1 University of California, Santa Barbara Climate Hazards Center, Santa Barbara, USA; 2 US Geological Survey, Earth Resources and Observation Science Center, Sioux Falls, SD USA; 3 Karlsruhe Institute of Technology (KIT), Institute of Meteorology and Climate Research, Karlsruhe, Germany; 4 Science Systems and Applications, Inc and NASA Goddard Space Flight Center, Greenbelt, USA; 5 University School for Advanced Studies IUSS Pavia, Pavia, Italy; 6 Department of Engineering, University of Messina, Messina, Italy

**Keywords:** Atmospheric science, Hydrology, Environmental sciences

## Abstract

The Climate Hazards Center Infrared Precipitation with Stations (CHIRPS) data stream combines: (1) a high-resolution climatology, (2) thermal infrared (TIR) geostationary satellite observations, and (3) station observations. In the past, CHIRPS version 2 (CHIRPS2) has proven to be valuable for drought monitoring, hydrologic modeling, scientific studies and agricultural decision making. Version 3 (CHIRPS3) improves each of these components. The new version, CHIRPS3 extends to 60°S/N, adopts an improved variance-preserving TIR-to-precipitation estimation method, uses many more stations and station sources than the original CHIRPS2 product, and implements gauge-undercatch correction. In this paper, we evaluate the performance of satellite-only CHIRP3, CHIRP2, IMERG, PERSIANN- CCS, and GPI using high quality interpolated data in twelve regions with dense station coverage. CHIRP3 represents both the observed mean and variance more accurately than CHIRP2. A usage section in Morocco shows that CHIRPS3 better captures the observed rainfall variability when compared to CHIRPS2. This section also demonstrates how station data should be gauge-undercatch-corrected when validating CHIRPS3.

## Introduction

**Fig. 1:**
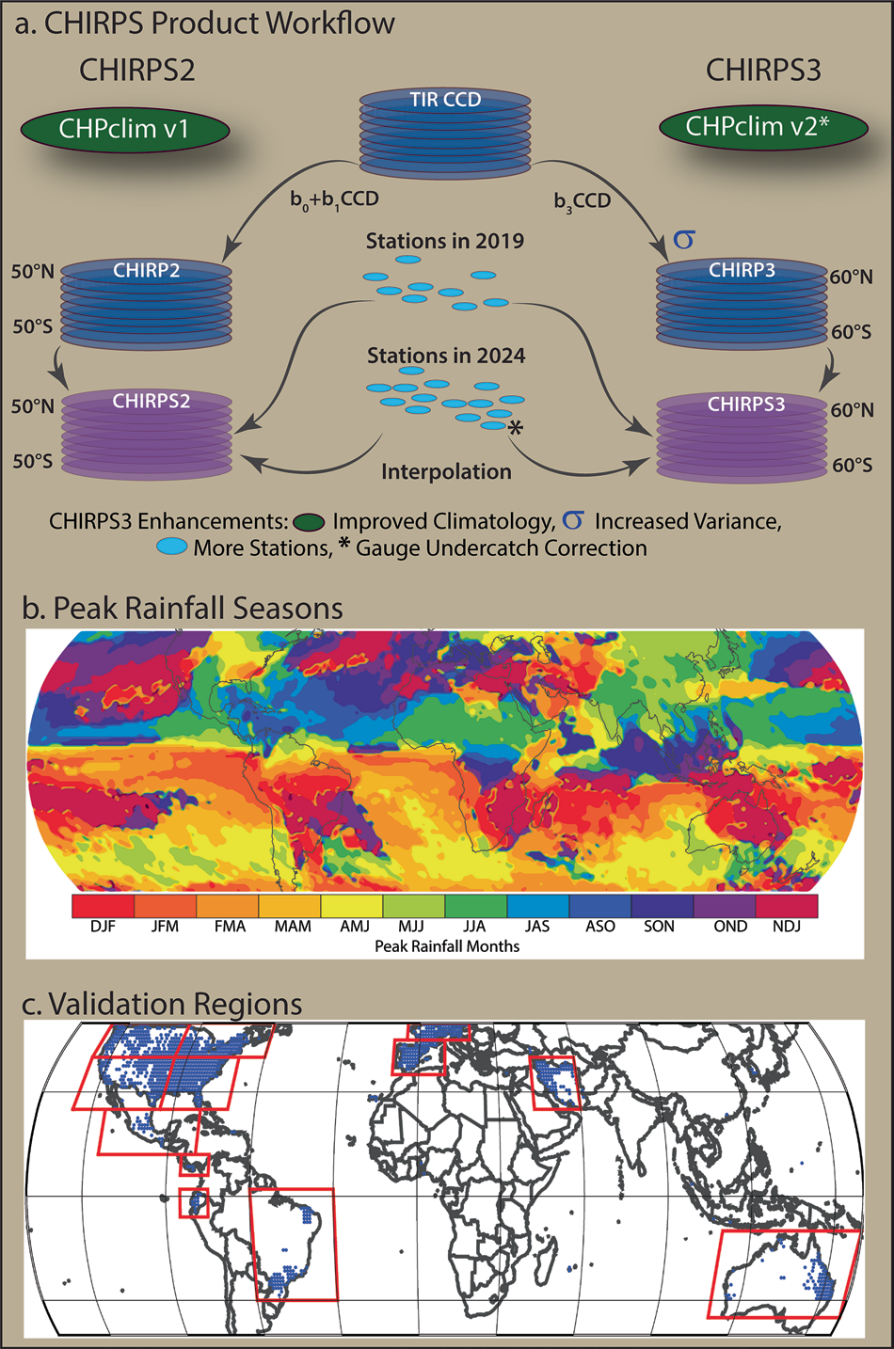
(**a**) A schema describing the three main components of CHIRPS2 and CHIRPS3 (satellite-enhanced climatology, TIR CCD precipitation estimates, and rain gauge observations), as well as the key improvements in CHIRPS3: an improved climatology, a variance-preserving CCD estimation procedure, many more stations and station observations, and a transition to gauge-undercatch correction. (**b**) Peak rainfall seasons used in the validation process. (**c**) REGEN validation locations and regions. Note that when calculating error statistics, only the well-instrumented sites—shown with blue dots—were used. Satellite-only precipitation estimates were validated with REGEN, hence there is no overlap with the REGEN station information.

High resolution, accurate, and routinely updated gridded precipitation data are essential for famine early warning^1^. Long records are especially important for evaluating the historical extremity of individual events, rainfall trends, and linkages with climate drivers. These datasets have helped humanitarian agencies and their scientific support teams identify areas with extreme rainfall or developing droughts, and drive crop and hydrological models for estimating impacts^2^. To support these types of applications, the University of California, Santa Barbara (UCSB) Climate Hazards Center (CHC), in collaboration with the U.S. Geological Survey and NASA SERVIR, developed version 1 of the Climate Hazards Center Precipitation Climatology (CHP_clim_ v1)^3^, the satellite-only CHIRP2 dataset, and the satellite and station-blended CHIRPS2 dataset^4,5^. The CHIRPS2 and CHIRPS3 products take advantage of many sources of information (Fig. 1a): a satellite-enhanced climatology (CHP_clim_), Thermal InfraRed (TIR) Cold Cloud Duration (CCD) precipitation estimates (CHIRP), and rain gauge observations (station data). The climatology benefits from the large number of long-term climate normals and the ability of mean satellite precipitation fields to represent gradients well in most poorly instrumented areas^3^. The fidelity of these mean fields helps the time-varying CHIRP resolve local terrain influences, and these fields are further enhanced with station data, leading to CHIRPS. As described in this Data Descriptor, each of these components have been improved in version 3 (CHIRPS3). The climatology is enhanced with more stations, and converted to gauge-undercatch corrected values. The satellite estimation algorithm is improved to better represent the variance of precipitation, and many more station sources and observations are added.

## Background – The CHIRPS Product and CHIRPS Community

CHIRPS3 was designed to retain the key characteristics of the CHIRPS2 products that drive its performance—a sophisticated climatology, satellite TIR, and station data blending procedure—while also enhancing several facets that may allow CHIRPS3 to better represent the severity of both droughts and pluvials (Fig. 1a). CHIRPS3 incorporates an improved climatology, satellite-only variance-preserving CHIRP3 estimates that cover an expanded geographic range (60°S–60°N), and the inclusion of many more stations and station observations. Please note that throughout this descriptor, CHIRP will refer to the satellite-based estimate, while CHIRPS refers to these fields blended with gauge observations.

The CHIRPS data products are designed to occupy a special temporal niche between two common groups of precipitation datasets. On one end are rapidly updated monitoring products, such as the Integrated Multi-satellitE Retrievals for GPM (IMERG)^6^, the Precipitation Estimates from Remotely Sensed Information using Artificial Neural Networks Cloud Classification System—PERSIANN-CCS^7^ (Hong *et al*. 2004), the Multi-Source Weighted-Ensemble Precipitation, version 2 (MSWEP V2)^8^. On the other end are climatological data records such as the station-only Global Precipitation Climate Center (GPCC) Full datasets^9^, or the satellite-gauge IMERG Final^6^, and Global Precipitation Climatology Project Version 3.2^10^. While the former prioritizes speed-of-access (reduced latency) at the potential expense of accuracy, the latter prioritizes the highest quality inputs available at a reduced frequency (increased latency). To provide both timeliness and high quality, the CHIRPS suite uses a two-stage set of products featuring a lower-latency preliminary product (CHIRPS Prelim) for operational monitoring, and a final, more intensely quality-controlled version for historical analyses (CHIRPS Final).

The preliminary CHIRPS product (CHIRPS Prelim) is created every pentad with a 2-day lag. Pentads break a month into five five-day accumulation periods, and a sixth pentad contains the last 3 to 6 days of the month. These pentads serve as the foundational time step of the CHIRPS products. These estimates have the same CHIRP inputs as for the final CHIRPS product, but have fewer station observations available for the blending process, and are quality controlled using only automated processes. This automation and use of best-available station data ensure the CHIRPS Prelim product is produced with relatively low latency, and at consistent intervals as required for operational agroclimatic monitoring and early warning systems.

During the third week of the month, CHIRPS Final for the previous month is produced using an enriched selection of station data, and more careful screening using automated steps, diagnostics, and visual inspections by the Climate Hazards Center (CHC) ‘Reality Checks’ team. The remaining quality-controlled station values are blended with the satellite-only CHIRP data to produce CHIRPS fields. These fields, accompanied by diagnostic information, are provided as daily, pentadal, dekadal, monthly, and multi-month precipitation totals. CHIRPS Final is similar in quality to the GPCC, IMERG Final, and GPCPv3.2, but at a shorter latency, and in some regions, especially in recent years, CHIRPS contains more station observations than all of these.

In addition to standard sources of global station data used in the aforementioned products, the CHC station archive is enriched by contributions from numerous partner agencies. This significantly enhances the available information—particularly in the Americas and Africa—where many countries have been underrepresented in other global datasets. Many partners contribute data to CHIRPS, and the resulting CHIRPS products can be regarded as a collaborative co-produced dataset, freely provided to support science, commerce and risk management. This co-creation involves the CHC team (https://chc.ucsb.edu/people), but also the many institutions that share the station data that enhances the CHIRPS products (Table 1).

**Table 1:** Agencies and institutions contributing data to support CHIRPS.

Source	Region
Meteorological Service of Canada	Canada
CEMADEN - Centro Nacional de Monitoramento e Alertas de Desastres Naturais	Brazil
Chile Met - Dirección Meteorológica de Chile (DMC)	Chile
Conagua - Comisión Nacional del Agua	Mexico
COPECO - Comité Permanente de Contigencias	Honduras
Deutscher Wetterdienst (DWD) - Germany National Meteorological Service	Germany
EMI -Ethiopian Meteorological Institute	Ethiopia
FSU - Florida State University	Africa
IDEAM - Instituto de Hidrología, Meteorología y Estudios Ambientales	Colombia
IMHPA - Instituto de Meteorología e Hidrología de Panamá	Panama
IMN - Instituto Meteorológico Nacional de Costa Rica	Costa Rica
INAM - Instituto Nacional de Meteorologia	Mozambique
INSIVUMEH - Instituto Nacional de Sismología, Vulcanologia, Meteorologia e Hidrología	Guatemala
INMET - Instituto Nacional de Meteorologia	Brazil
KIT - Karlsruhe Institute of Technology	Africa
KMA - Korea Meteorological Administration	South Korea
KMD - Kenya Meteorological Department	Kenya
MSD – Meteorological Services Department of Zimbabwe	Zimbabwe
NOAA Climate Prediction Center	Global
NOAA National Center of Environmental Information	Global
SASSCAL - Southern African Science Service Centre for Climate Change and Adaptive Land Management	Southern Africa
SEPA - Scottish Environment Protection Agency	Scotland
SISSA - System for southern South America	Southern S. America
SMN - National Meteorological Service	Belize
SNMA - Somalia National Meteorological Agency	Somalia

Arranged alphabetically.

To support agroclimatic monitoring and hazards outlooks, the CHC also produces a daily-updated 5, 10 and 15-day precipitation forecast dataset that is compatible with CHIRPS, using downscaled and bias-adjusted (quantile matched) NCEP Global Ensemble Forecast System (GEFS) precipitation products, as described in a Scientific Data paper^11^. The resulting stream of interoperable products (CHIRPS Final, CHIRPS Prelim, and CHIRPS-GEFS) provides a seamless information suite that extends from 1981 into the near-future. The CHC Early Estimates portal (https://chc.ucsb.edu/monitoring/early-estimates/info) provides access to these integrated products, as do several instances of the Early Warning Explorer^12^.

Numerous validation studies have found that the long period of record, low latency, low bias, and good performance of CHIRPS2 and CHIRP2 make the data well-suited for many early warning, crop monitoring, hydrologic modelling and science applications^13–26^. In an age of limited resources, these products can help efficiently allocate assets—such as water, fertilizer and food aid—to the fields and people who need it the most. These allocations can help maximize agricultural production and minimize human suffering and societal disruption. Despite its merits, some studies identify notable deficiencies in CHIRPS2. The first major issue is that CHIRPS2 tends to underestimate precipitation extremes, particularly during high-intensity events^27^. Another issue that has been identified is that in regions with complex terrain and high winds, CHIRPS2 underestimates precipitation due to gauge-undercatch—systematic errors in wind-affected rain gauges^15^. A third deficiency is that CHIRPS2 tends to produce spurious precipitation (i.e. drizzle—small, non-zero values that can inflate totals and wet-day counts). Collectively, these shortcomings can compromise the accurate representation of hydroclimatic extremes and variability. As described in Fig. 1a, and in the methods below, our ultimate development goal has been to create a new product that is similar to the widely-used CHIRPS2, but with an improved climatology, an enhanced variance-preserving CCD estimation algorithm, and the inclusion of many more stations.

## Methods

### CHIRPS Inputs

#### Climatology

CHIRPS3 uses gauge-undercatch corrected station data, and a new gauge-undercatchundercatch-corrected climatology which was produced using IMERG v6 Final, elevation data, and approximately 84,000 long-term average monthly gauge-undercatch corrected climate normals^28^. The development of this new climatology—the Climate Hazards Precipitation Climatology version 2 (CHP_clim_ v2)—is described in Funk *et al*.^27^. This paper describes the Climate Hazards IMErg with Stations (CHIMES) dataset. For more information, we refer readers interested in the new climatology to that article.

The CHIRPS modeling approach leverages the advatages of accurate, high resolution long-term mean fields to address the limitations caused by station data inconsistencies. In areas with sparse station data, there are often many more *in situ* observations from the past than from the present. Furthermore, the number of available station observations is typically declining over time due to a lack of maintenance, financial constraints, and poor reporting infrastructure. For example, in a large country like Tanzania, we might only have five or ten stations from last month, but have in hand hundreds of long-term average values from the 1990s or 1980s. The hundreds of long-term values can be used to produce a high-quality long-term mean field, which can then be used to enhance performance of real-time precipitation estimates, essentially by ‘borrowing’ spatial-temporal information from the past to help resolve environmental gradients between stations. Additionally, topography, seasonally-varying dominant wind directions, and other geographic features provide some aspect of ‘predictable’ typical spatial patterns that a good monthly rainfall climatology can resolve. This has advantages. For example, in mountainous or hilly areas in many tropical regions, orographic effects often result in more rainfall at higher elevations, especially when the atmosphere contains substantial amounts of water vapor.

The low bias provided by accurate mean fields reduces the disruptive impacts of changes in station data networks. When stations appear and disappear from observation networks, large differences from the climatological means can translate into large spurious non-random systematic shifts in precipitation. For example, large systematic differences between station observations and satellite-based estimates in Africa, combined with large decreases in the density of station observations, have been shown to produce spurious trends in blended satellite-station precipitation estimates^29^.

The 1991–2020 CHP_clim_ v2 monthly mean fields are produced using a combination of moving window regression and spatial interpolation to describe local gradient behavior^27^ (Fig. 1a). Monthly fields are disaggregated to smoothly-varying pentadal mean fields. CHP_clim_ v2 inputs include more than 84,000 gauge-undercatch-corrected climate normals from GPCC and CHC stations, elevation, mean monthly IMERG v6 Final data, and the CHP_clim_ v1^3^. The spatial component of the CHIRP—the satellite-only component of CHIRPS—is based on pentadal 0.05° CHP_clim_ long-term mean fields.

#### Time-varying geostationary satellite thermal infrared (TIR) observations

To derive the time-varying component of CHIRP3, the CHC team adopted a local TIR Cold Cloud Duration (CCD) calibration approach, such as those used in the Tropical Applications of Meteorology using SATellite and ground-based observations TAMSAT^30^ product and CHIRP2. Like CHIRP2, CHIRP3 relies heavily on two valuable archives of global inter-calibrated geostationary TIR imagery—the 1981–present GridSat B1 archive^31^ and the 2000–present Climate Prediction Center (CPC) archive^32^. CCD fields are combined with the CHP_clim_ v1 and v2 climatologies to produce the satellite-only CHIRP2 and CHIRP3 products. Next, station-enhanced monthly and pentadal CHIRPS products are then produced by blending in stations with CHIRP using a modified inverse distance weighting procedure^5^, which uses the local spatial correlation of the CHIRP data (refer to Section 2.2). Because the satellite-only CHIRP3 and CHIRP2 have low bias, in comparison to station data, variations in the stations reporting from month-to-month have relatively limited negative impacts on performance. Thus, information from long-term means, satellite CCD observations, and *in situ* measurements are combined in a transparent and consistent manner. Daily station blending is not performed because a) the spatial coherence of daily precipitation limits the spatial influence of the daily observations, b) the distribution of daily precipitation is extremely skewed, and c) the definition of a ‘day’ varies substantially based on the source dataset and country.

To calculate CCD, CHIRP3 uses TIR data to estimate the number of hours at each pixel, for every pentad, where the observed TIR was less than 235 kelvin (K). The CHIRP3 algorithm (Fig. 1a) uses a different calculation than the CHIRP2 algorithm to translate CCD to mm of rainfall. This change was adopted to reduce systematic errors that came from the intercept term in the CHIRP2 regression equation that translated pentadal CCD to rainfall in mm. These intercepts led to a tendency for CHIRP2 to underestimate precipitation variance, while also producing a high amount of small but non-zero pentadal estimates. In CHIRP2, these regressions were derived using global estimates from the NASA TRMM Multi-satellite Precipitation Analysis (TMPA)^33^. In CHIRP3, this translation from CCD to rainfall is derived more directly from the relationship between historical TIR observations and the CHP_clim_ v2. The algorithm is described in Section 2.2.

#### The CHC station archive

The CHIRPS products build on station observations from multiple sources (Table 1). In addition to global sources such as monthly Global Historical Climate Network version 4^34^, the daily Global Historical Climate Network^35^, the Global Telecommunication System archive, and the Global Summary of the Day dataset, CHIRPS benefits from the critical contributions provided by CHC field scientists stationed in Africa and Central America. The efforts from these scientists, along with the efforts of CHC personnel and collaborators in the United States, Africa, Central America, South America and Europe, has provided access to thousands of new stations in the developing world. In Africa, CHC archives have been augmented by contributions provided by the Karlsruhe Institute of Technology and Florida State University. The new Global Historical Climate Network version 4^34,36^ (GHCNv4) also provides an important new resource. The GHCNv4 contains many more station observations the previous GHCN version 3. A large (~72,000) set of gauge-undercatch corrected climate normals was provided by the Global Precipitation Climatology Centre (GPCC). National meteorological agencies in Mexico (Conagua), Ethiopia (EMI), Guatemala (INSIVUMEH), Panama (ETESA), Belize (NMS), Costa Rica (IMN), Honduras (CENAOS-COPECO) and the Comite Regional de Recursos Hidraulicos have shared station observations. Along with these countries and in Somalia, Angola, Botswana, Namibia, South Africa, Zambia, Brazil, Colombia, and Panama, the CHC has acquired or is working towards the timely acquisition of station data for the purposes of enhancing gridded precipitation data that can support agricultural monitoring applications.

Between 2019 and 2024, the number of sources and number of routinely-updated stations assimilated by the CHC has increased dramatically for both monthly and sub-monthly time steps. Table 2 shows the routinely-updated station data used in the CHIRPS2 products. As of 2019, 12 sources regularly provided approximately 11,500 unique monthly stations that were incorporated into CHIRPS2 ‘Final’ data. Seven of these sources (9,500 stations) provided daily data that could be used to generate pentadal station values. Of these, approximately 7,000 stations were available at a (<2-day) latency sufficient for use in the CHIRPS Prelim pentad data.

**Table 2:** CHIRPS2 baseline precipitation stations by source.

Source and Citation	Region	Number	Daily?	Used in Preliminary CHIRPS2?
**GHCNv2**^55^	Global	1200		
**GSOD**^56^	Global	5,400	yes	
**GHCN Daily**^57^	Global	6,600	yes	
**GTS**^58^	Global	6,400	yes	yes
**FAO Swalim**^59^	Somalia	80		
**Conagua**^60^	Mexico	800	yes	yes
**Ethiopian Meteorological Institute**^61^	Ethiopia	110		
**Dirección Meteorológica de Chile**^62^	Chile	20	yes	
**IMHPA**^63^	Panama			
**INSIVUMEH**^64^	Guatemala	80	yes	
**SASSCAL**^64^	Southern Africa	35		
**IDEAM**^65^	Colombia	600	yes	
**Unique Stations in 2019**		Monthly	Final Pentad	Preliminary Pentad
		**11,483**	**9,527**	**6,970**

These stations are representative of the routinely updated CHIRPS2 station inputs in 2019. Also noted are whether the data had daily data, and whether these daily data were available rapidly enough so as to be used in CHIRPS2 preliminary estimates.

Table 3 provides a similar summary for of stations for CHIRPS3 in late 2024. CHIRPS3 Final data is supported by 29 sources, which provide approximately 21,500 unique station observations. Twenty-two of these sources provide daily station observations, resulting in 18,000 unique pentadal station observations. Of these, 12,000 unique observations are available for use in CHIRPS3 Prelim pentad data. In both the Final and Prelim; the number of stations is e almost twice as many in 2024 as in 2019. A list of stations used in the monthly CHIRPS and the station observations are available at https://data.chc.ucsb.edu/products/CHIRPS/v3.0/.

**Table 3:** CHIRPS3 2024 precipitation stations by source.

Source	Region	Number	Daily?	Used in Preliminary CHIRPS3?
**GHCNv4**^36^	Global	12,000		
**GSOD**^56^	Global	5,400	yes	yes
**GHCN Daily**^57^	Global	6,600	yes	yes
**GTS**^58^	Global	6,400	yes	yes
**MCDW**^66^	Global	2,000		
**FAO Swalim**^59^	Somalia	80	yes	
**Ethiopian Meteorological Institute**^61^	Ethiopia	110		
**Meteorological Services Department of Zimbabwe**^67^	Zimbabwe	18		
**3D-PAWS - Kenya Meteorological Department**^68^	Kenya	36	yes	yes
**CEMADEN**^69^	Brazil	2,300	yes	yes
**Dirección Meteorológica de Chile**^62^	Chile	20	yes	yes
**Conagua**^60^	Mexico	800	yes	yes
**COPECO**^70^	Honduras	50	yes	
**IMHPA**^63^	Panama	80	yes	
**IDEAM**^65^	Colombia	600	yes	yes
**IMN**^71^	Costa Rica	13	yes	yes
**INAM**^72^	Mozambique	25		
**INSIVUMEH**^64^	Guatemala	90	yes	yes
**INMET**^73^	Brazil	550	yes	
**KMA**^74^	South Korea	700	yes	yes
**SISSA**^75^	Southern S. America	550	yes	yes
**NMS**^76^	Belize	15	yes	
**INMET**^73^	Brazil	550	yes	
**National Meteorological Agency**^77^	Somalia	28	yes	
**SASSCAL**^64^	Southern Africa	32		
**Meteorological Service of Canada**^78^	Canada	842	no	
**SEPA**^79^	Scotland	48		
**DWD**^80^	Germany	1,872	yes	yes
		Monthly	Final Pentad	Preliminary Pentad
**Unique Stations in 2024**		21,528	17,882	12,290

These stations are representative of the routinely updated CHIRPS3 station inputs in December of 2024. Also noted are whether the data had daily data, and whether these daily data were available rapidly enough so as to be used in CHIRPS2 preliminary estimates.

**Fig. 2:**
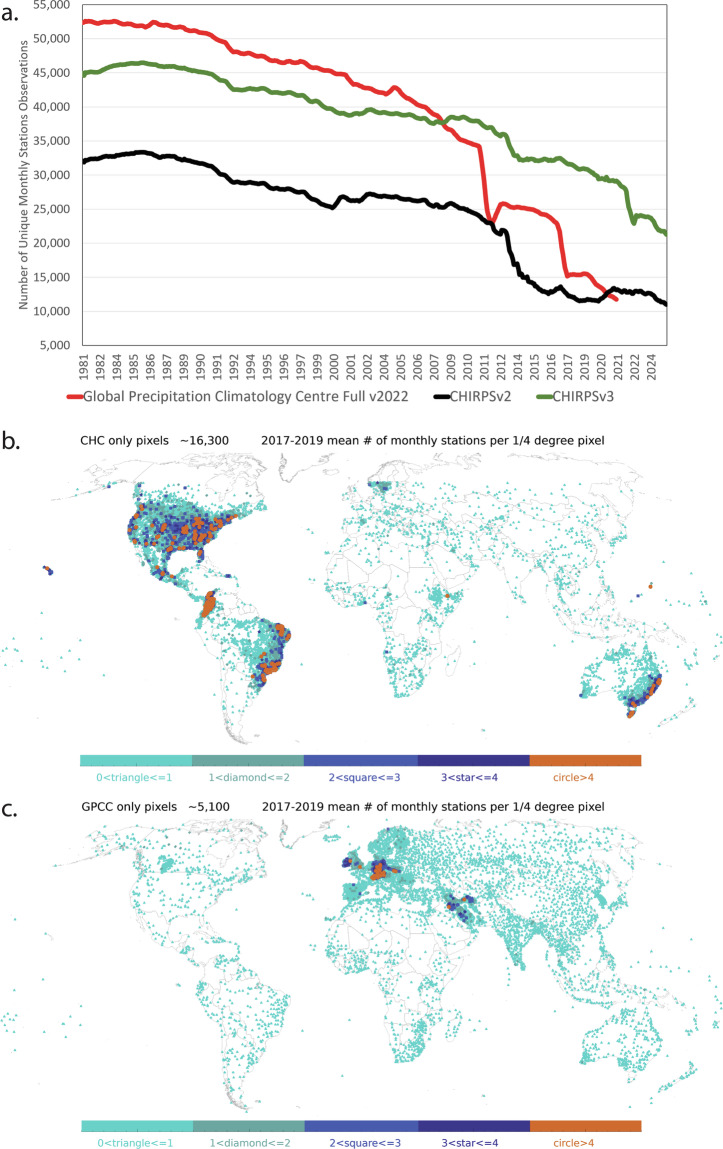
(**a**) Time series of CHC and GPCC stations counts. (**b**) Map of 2017–2019 average monthly station counts for the CHC v3 station archive, for 0.25° grid cells. (**c**) Same for GPCCv2022.

Figure 2a and Table 4 describe the temporal evolution of the CHC precipitation archives, in comparison with the stations included in the Global Precipitation Climatology Center (GPCC) Full 2022 product^37^. Over time, all archives tend to exhibit a concerning decline in the number of available station observations. CHIRPS2 has fewer stations than the GPCC Full product. The GPCC Full product, however, exhibits a large decrease in the number of stations in 2011 and 2017, such that the number of stations in 2020 (~12,000) is only one-third of the number available in 2008, and not much more than the number available in the GPCC monitoring product (~10,000). From 2012 on, the CHIRPS3 archive has substantially more station observations, with about 30,000 stations in 2020 compared to 12,000 in the GPCC Full product.

**Table 4:** Comparison showing the number of unique, global, CHIRPS2, CHIRPS3, GPCC Full, and GPCC First Guess archives, for selected years.

Product	CHIRPS2	CHIRPS3	GPCC Full v2022	GPCC First Guess
Latitude range	50S-50N	60S-60N	90S-90N	90S-90N
2004	26,145	39,240	42,207	8,096
2008	25,732	38,979	36,486	8,347
2012	21,223	35,996	25,737	9,210
2016	12,926	32,947	21,801	9,571
2020	12,901	30,186	11,959	9,675
2024	11,483	21,528		9,585

The CHIRPS archives include both routinely updated archives (Tables 1 and 2), as well as static inputs.

Figure 2b,c summarize the average number of unique stations in the CHC and GPCC archives, in 0.25° grid cells, over the 2017–2019 time period. In each map, grid cells with one observation on average are shown with cyan triangles. Those with two 3 are identified with green-blue diamonds. Blue and dark blue squares and stars denote areas with, on average, thee or four observations. Finally, orange circles denote grid cells with five or more observations, on average. The GPCC has relatively more stations in Europe, parts of Asia and parts of Africa, the CHIRPS3 tends to have more stations in the United States, Latin and South America and Australia.

#### Gauge-undercatch correction

Gauges in windy areas with complex terrain are known to under-observe ‘true’ precipitation. In complex terrain, winds give hydro-meteors horizontal velocity, which can lead precipitation observations to systematically underrepresent precipitation totals. This, in turn, can lead to underestimates in runoff and streamflow^15,38^. To address this concern, and to align with similar products being produced by the GPCC^38^ and NASA^10,39^, the CHP_clim_ v2 and CHIRPS3 employs gauge-undercatch correction. Early work on this topic yielded a widely-used set of ½ ° monthly estimates^40^. A more recent and complex analysis has been developed by the GPCC based on station data^28^. The gauge-undercatch correction that is applied to the station reports used in CHIRPS3 and the CHP_clim_ v2 make CHIRPS3 products modestly wetter than CHIRPS2 in most areas. Our monthly and pentadal station observations are modified by the Legates-Willmott correction factors^40^, and the CHP_clim_ v2 is based on gauge-undercatch corrected station data.

When comparing CHIRP3 and CHIRPS3 to independent station data, it is important to account for the gauge correction adjustment. An example validation conducted in Morocco is presented in the usage section below.

#### CHIRP3

Several objectives were considered when developing the CHIRP3 algorithm. An expanded latitude range and a transition to gauge-undercatch corrected estimates were straightforward. More challenging was an interest in producing a product that yielded more variance, but maintained low bias and high levels of similarity with CHIRP2. Prior research identified a systematic under-representation of precipitation variance in CHIRP2^21,27^. This included a spurious tendency towards non-zero drizzle in dry areas—as well as a tendency for both precipitation extremes and precipitation deficits to be underestimated. Essentially, the CHIRP2 algorithm can be expressed as a linear regression estimate, ‘trained’ using NASA TMPA^35^ data and the CHP_clim_ v1 (Fig. 1a). In general, if a 3D cube of CHIRP2 is estimated via this approach, then we can write: CHIRP2 = b_0_ + b_1_CCD. The expected total variance of CHIRP2 will be Var(CHIRP2) = Var(b_0_) + Var(b_1_CCD). Hence, the use of a constant non-negative intercept term in pentadal CHIRP2 values ‘soaks up’ some of the variance, leading to a systematic under-representation of the variance. While hard to identify in data-sparse regions, this issue became apparent in evaluations within data-rich regions^30^.

#### CHIRP3 CCD-to-precipitation slope fields

A foundational method of the CHIRPS procedure is identifying a relationship for converting Thermal InfraRed (TIR) Cold Cloud Duration (CCD) estimates to an estimate of precipitation. Over the past few years, numerous experiments with more complicated algorithms—based on quantile-matching and non-linear functions of CCD—were explored. These tended to increase the bias and mean absolute error of the candidate CHIRP3 fields, when compared against validation data, and thus were not selected. Eventually, these explorations led to the following relationship being selected for the slope term in the CHIRP3 algorithm.

1 $${b3}_{x,y,{pen}}=\frac{=\bar{{{CHP}}_{x,y,z}}+6\,{mm}}{\bar{{{CCD}}_{x,y,z}}+2\,{hours}}$$

The CHIRP3 slope value (b3) is a function of the pentadal CHP_clim_ v2 value ($\bar{{{CHP}}_{x,y,z}}$) and the mean pentadal CCD value ($\bar{{{CCD}}_{x,y,z}}$). The x,y,z in these equations represent spatial location and time. $\bar{{{CCD}}_{x,y,z}}$ in Eq. 1 represents the average number of hours in a pentad for which the TIR observations were colder than 235 K. 235 K is the most widely used CCD threshold. $\bar{{{CHP}}_{x,y,z}}$ is the local CHC climatology value, in mm per pentad. Constants are added to the numerator and denominator so that as the precipitation and CCD mean go to zero, the slope value relaxes to the GOES Precipitation Index^41^ rate of 3 mm per hour. Thresholds were also applied to b3, constraining the range of possible values to extend from 0.3 mm hour^−1^ to 25 mm hour^−1^.

Even when the CHP_clim_ v2 was zero, or near zero, it was considered valuable to have a non-zero b3. A non-zero slope enables CHIRP3 to estimate precipitation during abnormally-timed events, such as late-in-season storms, and in arid to semiarid regions, where extreme localized rains or impacts from cyclones can still occur. In CHIRP2, estimated amounts were more closely tied to variations from the background climatology (mean). If the background mean field was very low, CHIRP2 had a tendency to underestimate precipitation magnitudes in rare but extreme events.

#### The CHIRP3 algorithm

The following equation shows the calculation of the ‘first-cut’ CHIRP3.

2 $${{CHIRP}3}_{{first}}={b3}_{x,y,{pen}}{{CCD}}_{x,y,{pen}}$$

The ${{CHIRP}3}_{{first}}$ in Eq. 2 is produced by scaling the pentadal CCD by the CCD-to-precipitation slope fields. While evaluations of the pentadal ${{CHIRP}3}_{{first}}$ precipitation performed well, statistically, there were places where dry-season precipitation estimates were too high. Non-precipitating stratus clouds produced non-zero CCD values. The associated precipitation estimates, in some areas, like the Sahel in boreal winter, tended to be too high. In other areas, where precipitation was not captured well by the CCD (i.e. some tropical warm rain regimes or extra-tropical regions), ${{CHIRP}3}_{{first}}$ underestimated seasonal precipitation. To address these inaccuracies pentadal per-pixel ‘nudge’ coefficients were calculated from the difference between the CHP_clim_ v2 and the 2001–2022 mean ${{CHIRP}3}_{{first}}$ precipitation. The 2001–2022 period was selected to reflect the beginning of the CPC TIR dataset.

3 $${n}_{x,y,t}={\overline{{CHP}}}_{x,y,t}-\bar{{B3}_{x,y,t}{{CCD}}_{x,y,t}}$$

The offset factors described in Eq. 3 are combined with the CCD values and slope coefficients, to provide a second set of CHIRP3 estimates (Eq. 4).

4 $${{CHIRP}3}_{{second}}={{n}_{x,y,{pen}}+b3}_{x,y,{pen}}{{CCD}}_{x,y,{pen}}$$

Despite the correction procedure, non-physical precipitation estimates still occasionally appeared over some desert areas due to high cold stratus clouds that met the 235 Kelvin (K) CCD criteria in areas with very limited total columnar water vapor, such as the Sahara Desert in boreal winter. To screen these values, a dry mask (${m}_{x,y,t}$) was developed, based on the following criteria. For each pentad of the year, areas were identified where CHP_clim_ v2 was less than 0.5 mm. These areas were found to be very similar to where IMERG v7 Final and GPCC data showed very infrequent precipitation. Experimentation showed that this threshold did a good job at reducing the false, unseasonal precipitation estimates while also maintaining the ability of CHIRP3 to capture impactful events. For each pentad, an initial 0/1 dry mask was created, based upon whether the CHP_clim_ v2 was less than 0.5 mm. A sieve function was applied to regionally smooth the mask, resulting in values that smoothly transition from 0 to 1. A scaling term “m” was then created for masked areas (Eq. 5). This term estimates the degree to which the ${{CHIRP}3}_{{second}}$ precipitation estimate (using the slope term and nudge factor) historically overestimated precipitation, compared to CHP_clim_ v2 (${\overline{{CHP}}}_{x,y,t}$). In the locations that meet the dry masking criteria, estimates are scaled to smaller values. The ${{CHIRP}3}_{{second}}$ mean was calculated between 1981 and 2022. The epsilon value was set to 0.1 mm.

5 $${m}_{x,y,t}=\frac{{\overline{{CHP}}}_{x,y,t}+\varepsilon }{{\overline{{CHIRP}3}}_{{second},x,y,t}+\varepsilon }$$

The dry mask (d) was then combined with the bias adjustment fields (m) to generate the final CHIRP3 values (Eq. 6).

6 $${{CHIRP}3}_{x,y,t}={d}_{x,y,t}{m}_{x,y,t}{{CHIRP}3}_{{second}.x,y,t}+(1-{d}_{x,y,t}){{CHIRP}3}_{{second},x,y,t}$$

#### Combining GridSat B1 and CPC CHIRP3 records

While the half-hourly 2001–2022 CPC TIR record was the primary foundation for the development of CHIRP3, the 3-hourly GridSat B1 TIR archive was used to estimate the CHIRP3 values between 1981 and 2000. Because the B1 TIR data is 3-hourly, compared to the CPC ½-hourly observations, there are systematic differences in the CHIRP estimated from their differing CCD values. To account for these differences, multiplicative adjustment ratios were developed. The first step in this adjustment process involved using the CCD-to-precipitation slopes, nudge factors and dry mask fields described above to produce a first-cut set of 1981–2022 CHIRPS3 values based on the B1 data. The ratio of 2001–2022 pentadal means from the CPC and B1 CHIRP3 were then calculated, with a 7 mm constant in the numerator and denominator. These ratios were constrained between 0.25 and 1.75, and used to adjust the 1981–2000 B1 CHIRP3. The mean bias ratio was 1.11 mm mm^−1^. A value over 1 was expected, because less frequent sampling (3-hourly versus ½ hourly), tends to produce lower CCD values. The standard deviation of the bias ratios was 0.14 mm mm^−1^.

#### Filling pentads with missing TIR data using ERA5 Reanalysis

Due to gaps in geostationary satellite coverage, which varies by region and historical period, the satellite-only precipitation estimate—CHIRP—is sometimes discontinuous. A minimum value threshold is applied to the TIR data to identify erroneous data values. In such cases, where there is missing or erroneous TIR data, CHIRP was filled using unbiased ECMWF Reanalysis v5 (ERA5) precipitation data (the preliminary version of ERA5 made available about 5 days after real time). According to the ERA5 documentation, changes between the real time and final reanalysis fields are very rare. It should be noted that precipitation observations are not directly assimilated during the production of ERA5. Rather, precipitation is a forecast variable that is produced by the model physics during each assimilation cycle, and is subject to the biases and errors in the model.

This gap-filling is performed at the pentad timescale and is also reflected in the corresponding dekad and monthly accumulations. This applies to CHIRP and CHIRPS data from 1981–2022. Missing or erroneous TIR data from 2023 onward appear as −9999 in both the CHIRP and CHIRPS products. Historically, there are notable gaps in geostationary satellite coverage of certain regions that influence CHIRP, such as large coverage gaps in the early 1980s over Africa and a long coverage gap over part of Central Asia through 1997. Overall, the frequencyof missing CHIRP3 over land values was 20%, 4%, 0.5% and 0.4% in the 1980s, 1990s, 2000s, and 2010s, respectively. Due to CHIRPS being a blend of CHIRP estimates and *in situ* station observations, the influence of filled data on CHIRPS also varies based on station density. Users engaged in dataset comparisons and trend assessments can learn of which time periods and regions are gap-filled within CHIRP by examining fill mask GeoTIFs, available for download at several periodicities at the following link https://data.chc.ucsb.edu/products/CHIRPS/v3.0/diagnostics/fillmaps/, and by visiting the CHC EWX Next Generation Viewer at https://ewx3.chc.ucsb.edu/ewx/index.html, under CHIRP v3.0.

To address the question of how much model-generated precipitation might influence trends or multi-decadal variability, we carried out an analysis of ERA5 versus CHIRP3 bias between 1981 and 1989 (the 1980s) and between 2010 and 2019 (the 2010s). This was calculated as ABS(Mean(ERA5/CHIRP3)_2010s_ - Mean(ERA5/CHIRP3)_1980s_). Means were taken over all the pentads with valid TIR data. These values ranged up to 30%, with largest values in Central Africa and Central Asia. This map represented the amount of potential absolute percent bias, if all CHIRP3 data were missing in the 1980s.

The percent bias map was then multiplied by a map containing the fraction of missing pentads in the 1980s. Note that beginning in the 1990s the frequency of missing TIR data was very low. In poorly observed areas, around 40% of the TIR pentads were missing, though there is an area in northern Asia with no data. The product of the absolute percent bias map and the frequency of missing data map was quite low, on average. The mean value was 2.3% Note that 88% of pixels have an absolute percent bias of less than 5%, and 96% have an absolute percent bias of less than 10%. There are some areas where absolute percent bias is greater than 10% in Central Africa and Central Asia. There are also areas of northern central Asia that are completely lacking in TIR data. Decadal variations and trends would likely be quite uncertain in the CHIRPS products in these regions, given the large potential changes in ERA5 over time.

#### CHIRPS3 station blending method

The CHIRPS3 station blending process is based on a modified inverse distance weighting procedure. Several innovations from our experience with CHIRPS2 were adopted that accelerate and improve the blending process. The most interesting of these innovations is that the procedure uses empirical estimates of the precipitation decorrelation structure. These estimates are derived from the IMERG v6 Final dataset. Experimentation was also carried out using the latest IMERG v7, but isolated pockets of very low spatial correlation in v7 suggested that they might be less suitable to estimate precipitation decorrelation slopes.

The spatial correlation process begins by creating a 2001–2023 ‘cube’ of monthly IMERG Final values. The temporal correlation between IMERG values at a given grid cell and a grid cell from some distance away provided a measure of the local decorrelation slope. Our analysis used the values from 72 surrounding grid cells, evenly spaced at a distance of 1.5°, to estimate an ‘isotropic’ correlation value. This correlation is then converted to a decorrelation slope (${b}_{d}$), with units of correlation per km, by dividing the correlation by the distance to the 72 neighbors, in km. Assuming that the correlation for co-located values is 1 (i.e. that there is no geostatistical ‘nugget’ effect), then the expected correlation at some distance (d) will be $r=1-d{b}_{d}$. This slope value varies by month and location, and is used in two ways. First, it is used to assign inverse distance-based weights to a set of five nearest station observations, with the i^th^ weight (${w}_{i}$) proportional to Eq. 7:

7 $${w}_{i}=\frac{1.0}{{\left(1-{d}_{i}{b}_{d}\right)}^{2{\prime} }}$$

or

$${w}_{i}=0\,{if}\left(1-{d}_{i}{b}_{d}\right) < 0$$

where ${d}_{i}$ is the distance between each location and the i^th^ station. Following standard inverse distance weighting procedure, the five weights are scaled to sum to one. The ratios between the five nearest station values and the CHIRP3 are then calculated ($r$), and the weighted sum of these ratios is used to produce an adjusted CHIRP3 estimate (Eq. 8). Ratio values greater than 3 are capped at 3, to reduce the potential impact of erroneous station values.

8 $${{CHIRP}3}_{{adj}}={(w}^{T}r){CHIRP}3$$

Now, we have two estimates at each location, the satellite-only CHIRP3, and the station adjusted CHIRP3. Next, combining these two estimates, the final CHIRPS blending procedure is applied. This procedure combines the two estimates, using estimates of the associated correlations. The correlation between the CHIRP3 data and ‘true’ precipitation is assumed to be 0.5, a value based on empirical analyses. The correlation value for the adjusted CHIRP is based on the distance to the closest neighboring station (${d}_{\min }$), ${r}_{{adj}}=\,\left(1-{d}_{\min }{b}_{d}\right),{or}0{if}\left(1-{d}_{\min }{b}_{d}\right) < 0$. Finally, these correlation assumptions are used to assign weights and calculate CHIRPS3 (Eq. 9).

9 $$\alpha ={{r}_{{adj}}}^{2}{\left({{r}_{{adj}}}^{2}+{0.5}^{2}\right)}^{-1},\beta =1-\alpha $$

$${CHIRPS}3=\alpha {{CHIRP}3}_{{adj}}+\beta {CHIRP}3$$

This process is designed to limit the influence of individual stations to a reasonable spatial range, ensuring that no single station disproportionately affects the surrounding grid cells. It is important to note, however, that the CHIRPS3 blending procedure is not an exact interpolation method. Even when a station is located within a 0.05° grid cell, the procedure does not fully reproduce the station’s observed value. This is because if we assume that ${r}_{{adj}}\,$= 1, Eq. 9 will yield an $\alpha =0.8$ and a $\beta =0.2$. These weights reduce the ‘bulls-eye’ effect associated with the interpolation of station data, and reflect the geostatistical ‘nugget’ effect—the fact that two nearby stations will not be perfectly correlated over time. As a result, the final estimate is a weighted combination of the satellite and station data, rather than a direct substitution of the station value.

#### Estimates of CHIRPS variance explained (R^2^)

The weights from Eq. 9 (α, β) can be combined with estimates of the variance explained by the station-enhanced CHIRPS (${{r}_{{adj}}}^{2}$) and satellite-only CHIRP3 (0.5^2^).

10 $${R}^{2}=\alpha {{r}_{{adj}}}^{2}+\beta ({0.5}^{2})$$

The monthly variance explained maps are available as geoTiff images and pngs at https://data.chc.ucsb.edu/products/CHIRPS/v3.0/diagnostics/monthly.Rsquared.estimate/.

#### Daily CHIRPS disaggregation

In CHIRPS3 Final, two daily CHIRPS3 products are provided. They are based on the disaggregation of pentadal CHIRPS3 using either IMERG v7 Late^39^ or ECMWF ERA5 precipitation^42^. In each dissagregation case, the daily IMERG and ERA5 data are converted to ratios of the corresponding pentad total. These daily ratios are multiplied by the CHIRPS3 pentad total to produce daily CHIRPS3 estimates. When the ERA5 or IMERG pentad total is zero, but the CHIRPS3 pentad total is nonzero, the CHIRPS3 total is evenly distributed across the five days. It is important to note that the CHIRPS3 daily values are not independently derived; rather, they are disaggregated directly from the pentadal product. Consequently, the daily data are not updated on a day-to-day basis. Instead, they are generated in batches at the time of each pentad update. The IMERG-based CHIRPS3 daily archive is available since 2001 onward, while the ERA5-based CHIRPS3 daily product begins in 1981. Both are provided since the preferreed option may vary depending on the application. For CHIRPS Prelim, only IMERG-based daily disaggregations are provided. ERA5 has a larger (5-day) latency, and therefore is not available when CHIRPS Prelim pentads are made (2-day latency).

## Data Records

CHIRPS3 provides high-resolution (0.05° × 0.05°) gridded precipitation estimates over land for the global domain (7200 × 2400 pixels, 180°W to 180°E, 60°N to 60°S), Africa (1500 × 1600 pixels, 20°W to 55°E, 40°N to 40°S), and Latin America (1720 × 1900 pixels, 120°W to 34°W, 35°N to 60°S). These data can be found at the UCSB CHC CHIRPS3 webpage (https://www.chc.ucsb.edu/data/chirps3), where the CHIRPS3 data repository is also located. The CHIRPS 3 has the following Digital Object Identifier: 10.15780/G2JQ0P^43^ CHIRPS3 is in the public domain, as registered with Creative Commons, and is under a Creative Commons Attribution 4.0 International License. To the extent possible under the law, the Climate Hazards Center has waived all copyright and related or neighboring rights to CHIRPS3.

Two version of this data are available, preliminary (Prelim) and Final versions. The timely CHIRPS Prelim version is released two days after the end of a pentad on the 2nd, 7th, 12th, 17th, 22nd and 27th of the month. A longer latency CHIRPS Final version typically published in the third week of the subsequent month are available. Table 5 shows the update schedule for operational CHIRPS data. For the CHIRPS Prelim product, the data are available from January 2025 to present. The CHIRPS Final product has been back processed and is available from January 1981 to present. The CHIRPS datasets are distributed in different geospatial formats: GeoTIF, NetCDF, BIL, and COG, and each format has its own accompanying metadata.

**Table 5:** CHIRPS2and CHIRPS3 update schedule for the preliminary and final products.

Dataset	Time step	Update Schedule	Variable
CHIRPS-Prelim	Pentad	2nd, 7th, 12th, 17th, 22nd and 27th	Total (mm), Anomaly (mm), Z score
	Daily	After the pentad data are made	Total (mm)
	Dekad (sum of 2 pentads)	2nd, 12th, 22nd	
	Monthly (sum of 6 pentads)	2nd	
CHIRPS Final	Monthly	after 2nd week of the subsequent month	

CHIRPS3 is calculated at pentadal and monthly time scales, blending station data with CHIRP. The calculated pentads are rescaled such that the sum of six pentads equals the monthly CHIRPS. Pentadal CHIRPS data are used to make dekads and are downscaled using either IMERG v7 Late or ECMWF ERA5 to produce daily files. The monthly CHIRPS values are accumulated to make multi-month and annual totals (Table 6). These precipitation accumulations are available on the CHC data server. CHIRPS precipitation values are in total mm per time period, e.g., mm per day or mm per pentad. To support data interpretation and decision-making, precipitation anomalies (mm) and standardized anomalies (z-scores) are calculated for each time period based on the 1991–2020 climatology.

**Table 6:** CHIRPS3 data access, data records and formats.

Resource	Timeframe	Domain	Period	Format	Access
CHIRPS3 Dataset	Daily (ERA5-downscaled) Daily (IMERG v7 Late -downscaled)		January 1981 - present June 2000 - present	GEOTIFFS, COGS GEOTIFFS	https://data.chc.ucsb.edu/products/CHIRPS/v3.0/
	Pentad, Monthly	Global Africa Latin America	January 1981 - present	GEOTIFFS, NETCDFS, BILS, COGS GEOTIFFS, PNGS, BILS GEOTIFFS, BILS	
	Dekads	Global Africa Latin America	January 1981 - present	GEOTIFFS, NETCDFS, BILS GEOTIFFS, BILS, PNGS GEOTIFFS, BILS	
	2, 3, 4, 5, 6-monthly and annual	All domains	January 1981 - present	GEOTIFFS	
Early Warning Explorer (EWX) Next Generation Viewers	Global	Graphical interface featuring multiple datasets including historical CHIRPS3	January 1981-present	Time series for administrative and crop regions; images for user-defined extent.	USGS EWX https://earlywarning.usgs.gov/fews/ewx/index.html?region=gb UCSB CHC EWX (https://ewx3.chc.ucsb.edu/ewx/index.html)

For each new run of CHIRPS, the data undergoes rigorous quality control. This includes automatic screening of station inputs, and expert visual inspection through the reality checks process (R-Checks). The outputs of these quality control and diagnostic efforts are released alongside CHIRPS data and include a variety of resources: **1**. Maps of excluded stations containing false zeros or outliers **2**. CSV files that provide a summary of station locations used in the pentadal preliminary and monthly final CHIRPS3. **3**. GeoTif (.tif) files showing pentadal and monthly global station density **4**. Maps showing stations per country included in CHIRPS3 and **5**. CSV files containing monthly station values for all non-proprietary data in our database for that month **6**. Summary statistics that examine the new CHIRPS3 data against the entire CHIRPS time series and **7**. A report of the R-Checks on the CHC Wiki Page (https://wiki.chc.ucsb.edu/CHIRPS_Reality_Checks). Additional diagnostic products include fill maps that show where and when the CHIRP satellite-only rainfall estimates have been gap-filled using unbiased ERA5 reanalysis data, and monthly correction maps showing the Legates-Willmott correction factor used to adjust for systematic gauge measurement errors. The diagnostics products can be accessed at https://data.chc.ucsb.edu/products/CHIRPS/v3.0/diagnostics/ with a detailed description found on the CHC webpage at https://www.chc.ucsb.edu/data/chirps3/diagnostics. The Legates-Willmott corrections sub-directory contains global monthly one-degree correction factors. The CHC actively incorporates user feedback and evolving research needs by continuously developing new diagnostic products.

The CHIRPS3 products support a range of applications including agricultural advisories and anticipatory humanitarian interventions. To meet these needs, CHC produces Early Estimates—near real-time rainfall accumulation and anomaly maps covering periods from 1 to 18 pentads, and region-specific seasonal totals aligned with agricultural growing seasons of 3–6 months. The Early Estimates begin processing as soon as new Prelim or Final CHIRPS become available, and are generally available by end-of-day. Daily GEFS bias-corrected CHIRPS precipitation forecasts are also generated. These are available at https://data.chc.ucsb.edu/products/CHIRPS-GEFS/v3/. CHIRPS3 Early Estimates are available at https://data.chc.ucsb.edu/products/Early_Estimates/v3/.

## Technical Validation

### REGEN-based satellite-only validation results

In this section, we now present results based on the interpolated daily station dataset Rainfall Estimates on a Gridded Network (REGEN) produced by the University of New South Wales and the GPCC^44^. Developed to support analysis of sub-monthly rainfall variability, the 1° REGEN uses geostatistical interpolation (kriging) together with a background climatology. In this study, we use the REGEN grids from Frequent Rainfall Observations on Grids (FRoGs) archive^45^, which also provides additional sources of satellite-only precipitation estimates. Our analysis focuses on precipitation totals for the climatological wettest three-month season at each grid cell, as well as the 18 pentadal data values during that period (Fig. 1b). This corresponds to the three months of the year that are, on average, the wettest. Because the REGEN grids are a global product, it supports the examination of pentadal data in a set of 12 validation regions (Fig. 1c).

To maintain consistency with our previous CHIRPS2 paper, the wettest three-month period was based on CHP_clim_ v1. The 2001–2016 time period was used, because this period starts with the first full year of the Climate Prediction Center’s global TIR archive and ends with the last year in the REGEN archive. We compared REGEN data to several satellite-only estimates: CHIRP2, CHIRP3, IMERG v6 Late^6^, the GOES Precipitation Index (GPI)^41^, and the PERSIANN-CCS^7^. The PERSIANN-CCS estimates begin in 2003.

**Fig. 3:**
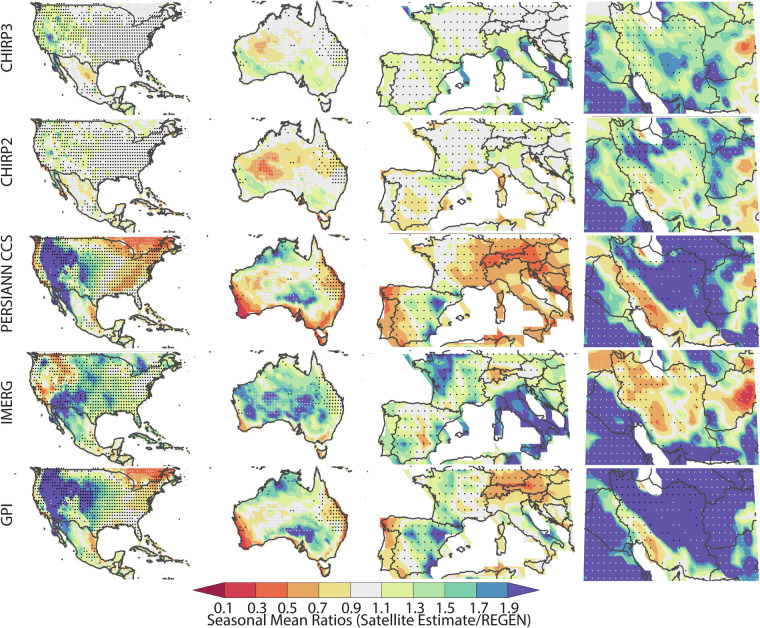
2001–2016 ratios of means (satellite estimate mean divided by REGEN mean), based on wet season totals for the United States and Mexico, Australia, Europe and Iran. These regions were selected because of their dense rain gauge networks. Small black dots denote cells with dense REGEN gauge data (average number of observations per > 7). Small gray dots denote cells with limited gauge observations (average number of observations per < 1).

**Fig. 4:**
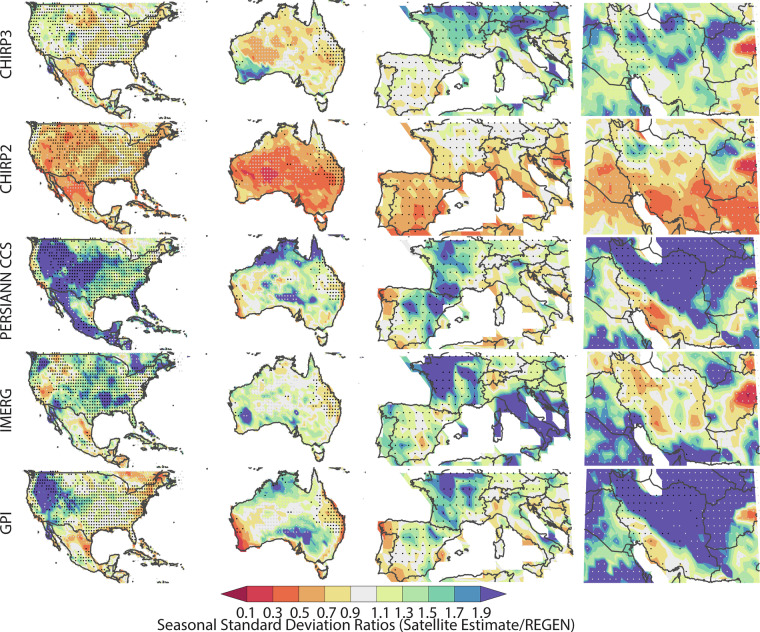
2001–2016 ratios of standard deviations (satellite estimate standard deviation divided by REGEN standard deviation), based on wet season totals. Small black dots denote cells with dense REGEN gauge data (average number of observations per > 7). Small gray dots denote cells with limited gauge observations (average number of observations per < 1).

**Fig. 5:**
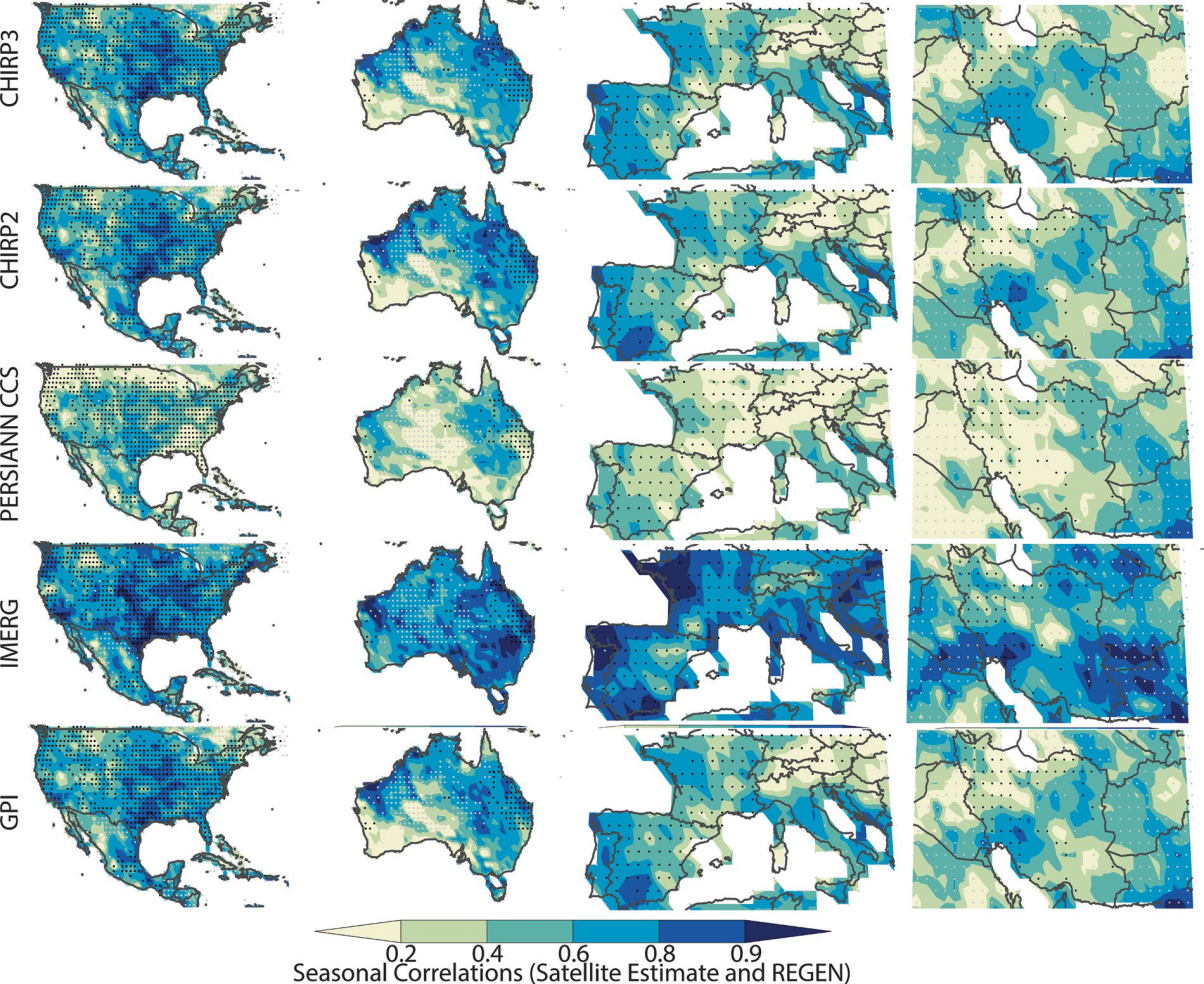
Correlations between 2001–2016 satellite and REGEN wet season totals. Small black dots denote cells with dense REGEN gauge data (average number of observations per > 7). Small gray dots denote cells with limited gauge observations (average number of observations per < 1).

**Fig. 6:**
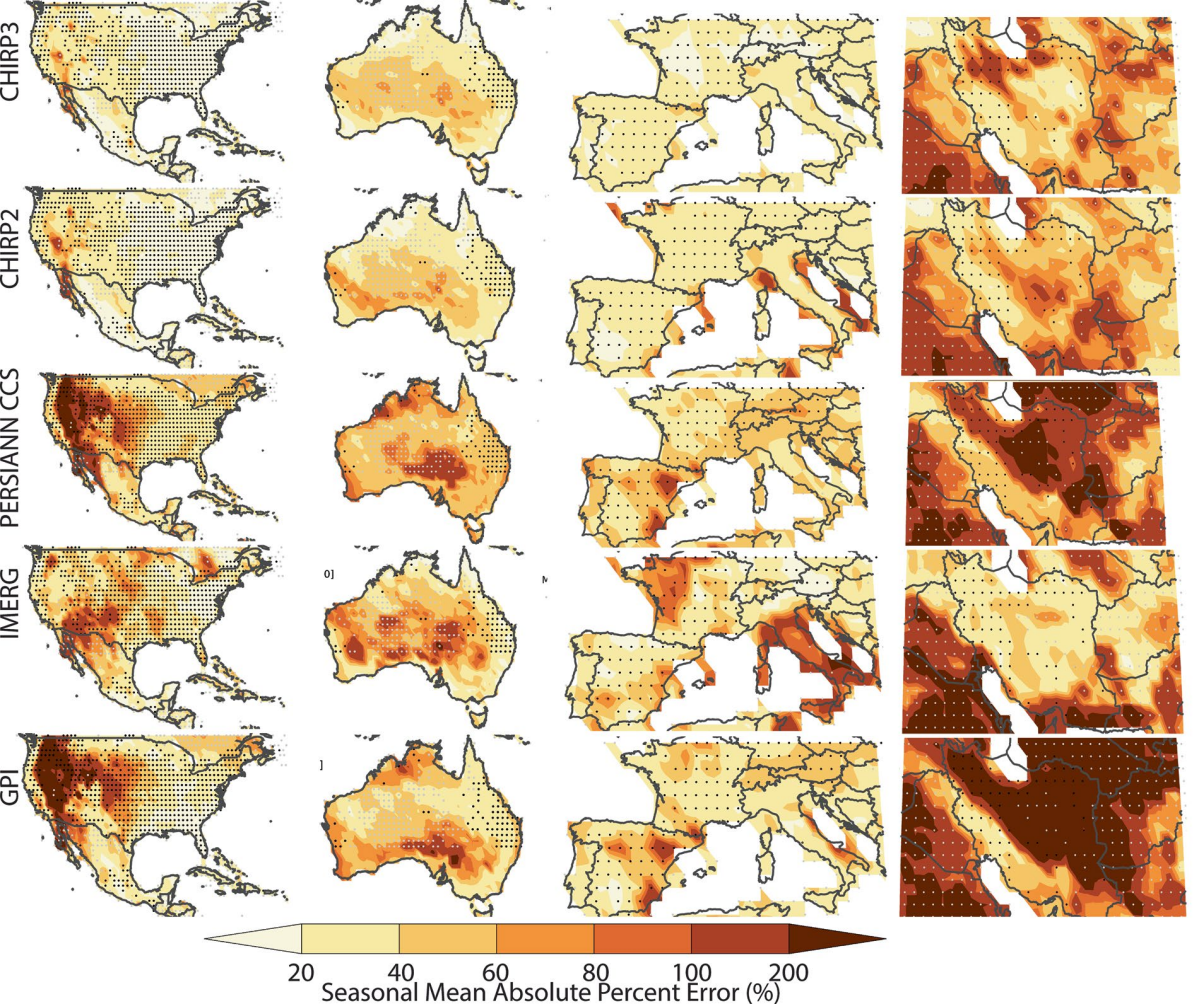
2001–2016 percent mean absolute errors, based on wet season totals. Small black dots denote cells with dense REGEN gauge data (average number of observations per > 7). Small gray dots denote cells with limited gauge observations (average number of observations per < 1).

By using the number of REGEN station observations, in each grid cell, for each day, we selected twelve well-gauged locations for detailed analysis (shown with blue dots in Fig. 1c and Tables 7–10). These very well-instrumented locations had, on average, at least seven rain gauge observations per day over the study period. This threshold was selected based on the tradeoff between spatial coverage and accurate REGEN estimates. When calculating the statistics shown in Tables 7–10, only these locations (i.e. the 1° grid cells identified with blue dots) were used. The four best-gauged regions were: 1) United States and Mexico, 2) Australia, 3) Europe, and 4) Iran (Figs. 3–6). Small black dots on these maps denote cells with dense REGEN gauge data (average number of observations per > 7). Small gray dots denote cells with limited gauge observations (average number of observations per < 1). The statistics in Tables 7–10 are based on the wet season pentadal data. The maps shown in Figs. 3–6 are based on the 3-month wet seasonal totals. Note that the statistics in the tables are based only on the grid cells with 7 or more observations, on average, every day.

**Table 7:** Wet season mean absolute percent bias and standard deviation ratios based on 18 pentadal data for 2001 through 2016 time period.

Mean Abs Bias (%)	CHIRP2	CHIRP3	IMERG	PERSIANN	GPI
SW US	9%	8%	45%	56%	54%
SE US	6%	5%	24%	19%	17%
NE US	6%	5%	19%	26%	25%
NW US	8%	5%	38%	76%	95%
Mexico	10%	10%	22%	20%	20%
Central America	28%	18%	21%	13%	18%
Ecuador	12%	3%	13%	37%	39%
Brazil	9%	4%	11%	22%	17%
South Europe	19%	7%	21%	33%	31%
North Europe	6%	4%	27%	33%	34%
Iran	35%	13%	42%	67%	126%
Australia	23%	16%	19%	34%	34%
**All Validation Cells**	**11%**	**7%**	**29%**	**42%**	**48%**
**Std Deviation Ratios**	**CHIRP2**	**CHIRP3**	**IMERG**	**PERSIANN**	**GPI**
SW US	54%	80%	114%	117%	102%
SE US	61%	78%	122%	85%	79%
NE US	63%	84%	116%	83%	85%
NW US	61%	91%	107%	100%	127%
Mexico	58%	73%	80%	85%	72%
Central America	43%	53%	59%	71%	52%
Ecuador	71%	134%	111%	85%	83%
Brazil	66%	88%	95%	101%	85%
South Europe	44%	81%	108%	69%	87%
North Europe	53%	101%	118%	60%	85%
Iran	52%	97%	69%	82%	135%
Australia	41%	63%	81%	64%	54%
**All Validation Cells**	**56%**	**82%**	**105%**	**86%**	**89%**

Statistics aggregated over one-degree grids cells with dense REGEN gauge networks. Mean percent bias values were calculated for each location, translated into absolute values, and then averaged.

**Table 8:** Percent deviations in 90^th^ percentile precipitation values from satellite datasets and the REGEN validation data.

Correlations	CHIRP2	CHIRP3	IMERG	PERSIANN	GPI
SW US	−25%	−4%	25%	34%	32%
SE US	−18%	−9%	20%	−13%	−8%
NE US	−15%	−4%	13%	−22%	−8%
NW US	−24%	2%	1%	43%	79%
Mexico	−17%	−12%	−3%	−3%	−8%
Central America	−40%	−31%	−31%	−22%	−31%
Ecuador	−19%	14%	3%	−28%	−26%
Brazil	−16%	−3%	−5%	4%	−4%
South Europe	−31%	−3%	3%	−8%	11%
North Europe	−24%	4%	20%	−34%	−13%
Iran	−7%	11%	−27%	12%	100%
Australia	−31%	−12%	7%	−21%	−21%
All Validation Cells	−21%	−3%	9%	3%	20%

Positive values indicate overestimation relative to REGEN, while negative values indicate underestimation.

**Table 9:** Wet season correlations and Mean Absolute Percent Error values based on 18 pentads of data for 2001 through 2016 time period.

Correlations	CHIRP2	CHIRP3	IMERG	PERSIANN	GPI
SW US	0.61	0.63	0.77	0.61	0.61
SE US	0.66	0.67	0.78	0.62	0.66
NE US	0.62	0.63	0.76	0.6	0.62
NW US	0.59	0.59	0.68	0.55	0.59
Mexico	0.6	0.62	0.65	0.6	0.6
Central America	0.44	0.45	0.48	0.27	0.44
Ecuador	0.43	0.41	0.6	0.41	0.43
Brazil	0.68	0.68	0.73	0.66	0.68
South Europe	0.59	0.62	0.75	0.6	0.59
North Europe	0.53	0.54	0.75	0.55	0.53
Iran	0.47	0.49	0.63	0.37	0.47
Australia	0.62	0.67	0.81	0.62	0.62
**All Validation Cells**	**0.6**	**0.62**	**0.74**	**0.58**	**0.6**
**Mean Abs Error (%)**	**CHIRP2**	**CHIRP3**	**IMERG**	**PERSIANN**	**GPI**
SW US	70%	69%	70%	92%	91%
SE US	56%	56%	56%	60%	59%
NE US	59%	60%	55%	62%	66%
NW US	61%	64%	63%	88%	102%
Mexico	49%	49%	50%	55%	52%
Central America	44%	41%	41%	46%	45%
Ecuador	50%	65%	51%	62%	66%
Brazil	50%	51%	46%	58%	55%
South Europe	76%	75%	62%	80%	81%
North Europe	64%	67%	58%	62%	70%
Iran	95%	90%	71%	106%	149%
Australia	66%	62%	52%	74%	70%
**All Validation Cells**	**61%**	**62%**	**57%**	**71%**	**74%**

Statistics aggregated over one-degree grids cells with dense REGEN gauge networks.

**Table 10:** Wet season probability of detection (POD) and bias scores.

Prob of Detection	CHIRP2	CHIRP3	IMERG	PERSIANN	GPI
SW US	20%	40%	70%	60%	60%
SE US	30%	40%	70%	33%	40%
NE US	30%	40%	60%	30%	36%
NW US	20%	40%	50%	55%	60%
Mexico	20%	30%	40%	40%	30%
Central America	0%	9%	10%	10%	0%
Ecuador	20%	40%	40%	11%	20%
Brazil	30%	40%	45%	45%	40%
South Europe	10%	40%	60%	40%	50%
North Europe	20%	40%	60%	20%	30%
Iran	30%	40%	30%	36%	60%
Australia	20%	45%	64%	36%	36%
All Validation Cells	20%	40%	60%	40%	50%
**Bias Score (%)**	**CHIRP2**	**CHIRP3**	**IMERG**	**PERSIANN**	**GPI**
SW US	0.40	0.90	1.60	1.70	1.80
SE US	0.50	0.70	1.40	0.78	0.80
NE US	0.60	0.90	1.30	0.60	0.82
NW US	0.40	1.00	1.10	1.91	2.50
Mexico	0.40	0.60	1.00	1.00	0.80
Central America	0.00	0.18	0.20	0.30	0.10
Ecuador	0.50	1.30	1.00	0.44	
Brazil	0.60	0.90	0.82	1.09	1.00
South Europe	0.20	0.90	1.00	0.90	1.20
North Europe	0.40	1.00	1.40	0.40	0.70
Iran	0.90	1.20	0.50	1.36	2.70
Australia	0.30	0.73	1.00	0.64	0.64
All Validation Cells	0.40	0.90	1.20	1.10	1.40

Statistics based on hits, misses and false alarms using the 90^th^ percentile of the pentadal REGEN precipitation. POD calculated as [hits/(hits + misses)] * 100%. Bias score calculated as [(hits + false alarms)/(hits + misses)].

Please note that this section is limited to satellite-derived precipitation estimates. Because we are selecting some of the best-sampled regions of the planet, comparisons between REGEN, CHIRPS2 and CHIRPS3 would be very favorable, but not really fair, because much of the same station data is used in all three products. Later sections of this study will provide a global comparison between CHIRPS3 and the GPCC Full product and a regional comparison using independent data in Morocco.

### Mean bias errors and standard deviation ratio results

By construction, the CHIRPS2 and CHIRPS3 data products are designed to have low bias errors. Additionally, a major improvement in CHIRP3 is much better performance than CHIRP2 in terms of accurate representation of the variance of wet-season precipitation. Overall, in the Americas, Australia, and Europe, the CHIRP2 and CHIRP3 bias fields are low. Figure 3 shows seasonal mean ratios, calculated using the seasonal mean of the satellite estimates and REGN. This strong performance is also found in IMERG v6 Late.

he PERSIANN-CCS and GPI datasets demonstrate substantially greater bias, particularly characterized by underestimation in the inter- mountain western United States and inconsistent performance-both under- and overestimation-across Iran.

To quantify numerically the products’ performance in terms of biases in the mean values, we have calculated the mean absolute value of the percent bias errors, based on the pentadal data in well instrumented 1° grid cells (Table 7). Some products, such as the PERSIANN CCS and GPI exhibited large negative and large positive biases (Fig. 3). Averaging percent biases, as opposed to the absolute value of the percent biases, tends to obscure large local deviations from the mean. The absolute mean bias errors, as reported in Table 7, were derived as follows:

11 $${absolute}\,{mean}\,{bias}\,{error}=100\ast \left({mean}\left(\frac{\left|{\mu }_{{obs}}-{\mu }_{{sat}}\right|}{{\mu }_{{obs}}}\right)\right)$$

Where ${\mu }_{{obs}}$ and ${\mu }_{{sat}}$ are means at individual grid cells. The CHIRP3 and CHIRP2 performance (7% and 11%) compares favorably with IMERG v6 Late, PERSIANN and GPI, which had mean values of 29%, 42% and 48%. across the regions.

The typical deviation of CHIRP3 from the true mean is approximately ±7%.

Considering the simplicity of the CHIRP3 algorithm, its strong performance in terms of standard deviation values is particularly notable (Fig. 4, Table 7). WhileCHIRP2 exhibits a problematic underestimation of the variance, especially in arid regions (Fig. 4), this problem is much reduced in the CHIRP3. The mean pentadal CHIRP2 standard deviations (Table 7) are only 56% of the REGEN standard deviations. This increases to 82% in CHIRP3. By this metric, on average, the IMERG, PERSIANN CCS and GPI also perform well, and in fact, moderately better (86–105%). The GPI and PERSIANN CCS appear to substantially overestimate variance in the northwestern United States and Iran.

### Evaluations of 90^th^ percentile pentadal precipitation intensities

Table 8 presents the ratios between the 90^th^ percentile pentadal satellite estimates, and the REGEN validation data, all expressed as percent anomalies. Overall, CHIRP3 performance is the best—just 3% below the REGEN values, on average. This compares well with the CHIRP2, which was 21% below the REGEN. PERSIANN CCS performed well according to this metric (+3%), followed by IMERG which modestly overestimated (+9%). The GPI tendency to overestimate was substantial (+20%).

### Correlation and mean absolute percent errors results

Figure 5 shows the correlation between wet season total precipitation in the REGEN and satellite precipitation datasets. Table 9 shows the correlation between wet season pentadal precipitation values. Not surprisingly, the four datasets that mainly rely on TIR data to estimate precipitation (CHIRP2, CHIRP3, PERSIANN-CCS, and GPI) perform quite similarly, and not all that well, with average pentadal correlations of 0.58 to 0.62. For IMERG, which benefits from microwave-based precipitation estimates, the average pentadal correlation was 0.74. It is important to note that high correlations at the pentad time scale are more difficult to achieve than for longer accumulation periods. Accordingly, correlations for wet season totals are higher (Fig. 5). Across the four study regions, IMERG shows the most spatially-consistent moderate-to-high correlation fields. The CHIRPS products provide a long period of record, extending back to 1981, but the performance of the satellite-only CHIRP2 and CHIRP3 varies from region to region. A TIR CCD-based, single temperature threshold method of measuring precipitation clearly has limitations, and lower correlations may be occurring in areas where precipitation is poorly associated with high cold cumulonimbus clouds and where there are substantial amounts of high, cold but non-precipitating cirrus clouds. Figure 6 and Table 9 display seasonal and pentadal Mean Absolute Percent Error (MAPE) results. MAPE is the MAE divided by the mean precipitation. Maps of wet season MAE divided by the REGEN mean (Fig. 6) show reasonable performance for PERSIANN CCS, IMERG, CHIRP2 and CHIRP3, with IMERG having the lowest overall pentadal MAE/mean ratios, followed by CHIRP2 and then CHIRP3 (Table 9).

### Wet-event probability of detection and bias scores

The observed REGEN 90^th^ percentile pentadal precipitation values were used to assess the satellite products’ ability to detect intense precipitation events. The events were classified as correct negatives, hits, misses and false alarms. For each 1° cell, the Probability of Detection (POD) was calculated as hits/(hits + misses) ×100. These results are presented in Table 10. IMERG and GPI^41^ performed well, with a POD of 60 and 50%. The GPI performance, however, is also related to substantial overestimation of 90^th^ percentile precipitation (+20%, Table 8). The PERSIANN-CCS and CHIRP3 had similar overall performance, with POD values of 40%. Among the evaluated products, CHIRP2 exhibited the lowest performance, with a probability of detection (POD) of 20%, indicative of its underestimation of standard deviation and 90^th^ percentile precipitation values.

Bias scores take into account hits, misses and false alarms, with the bias score equal to [(hits + false alarms)/(hits + misses)]. By this metric, CHIRP3 performed the best, with a bias score of 0.9. On average, CHIRP3 90^th^ percentile precipitation values were very close to the observations, which leads to similar numbers of misses and false alarms, and low bias scores. This may be contrasted with CHIRP2, which had more misses than false alarms, leading to a poor bias score of 0.40. IMERG, PERSIANN-CCS and GPI tended to modestly over-predict the frequency of wet extremes, leading to overall bias scores of 1.2%, 1.1% and 1.4%.

### An examination of CHIRP2 and CHIRP3 dekadal standard deviation scores in Ethiopia

**Fig. 7:**
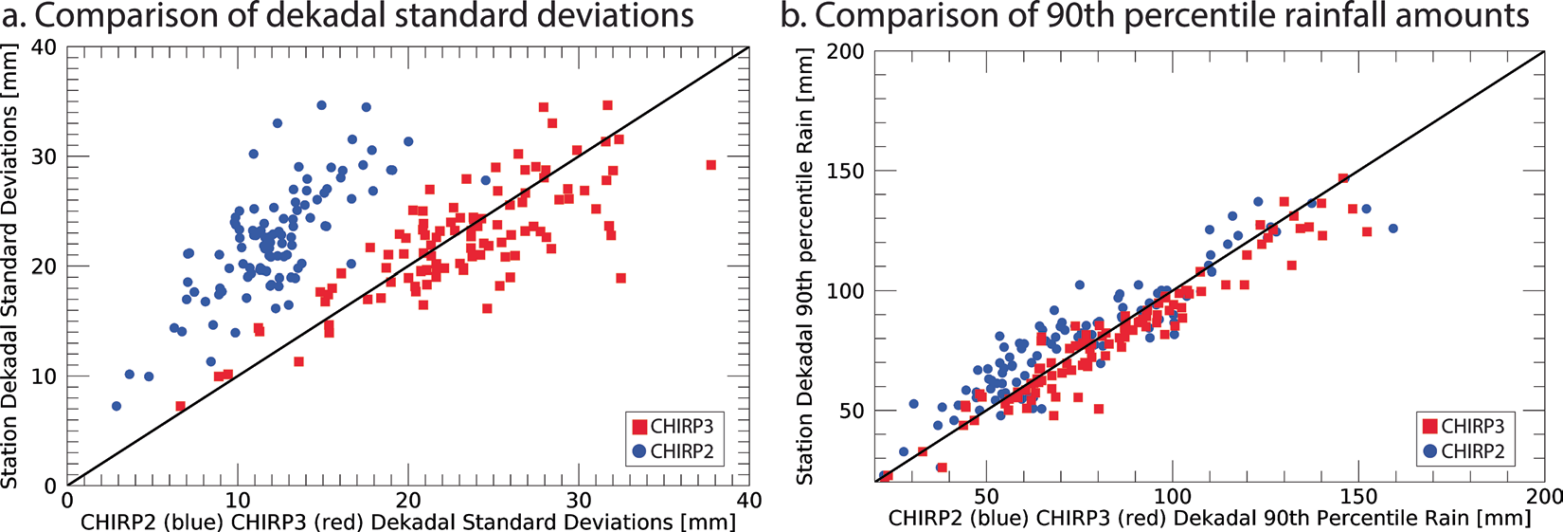
CHIRP2 and CHIRP3 validation analysis based on high-quality Ethiopian dekadal station data. Panel **a** shows a comparison of the average station and CHIRP2 and CHIRP3 1981–2019 dekadal precipitation standard deviations. Panel **b** shows a comparison of the 90^th^ percentile dekadal rainfall values, based on all the dekadal totals between 1981 and 2019. The CHIRP2 standard deviation and 90^th^ percentile R2 values were 0.54 and 0.85. The corresponding values for the CHIRP3 were 0.52 and 0.92.

The CHIRPS2 product^5^ and its climatology^3^ were originally developed to support food security in eastern Africa, where it has been shown to work well^46^. Long-standing collaborations between the CHC and the Ethiopian Meteorological Institute provide the CHC with good synoptic station coverage. This coverage, and the complex heterogeneous nature of precipitation in Ethiopia, provides a good opportunity to examine the performance of CHIRP2 and CHIRP3 variance. CHIRP2 and CHIRPS2 have been shown to have relatively low bias^46,47^ when compared to other products. Figure 7A illustrates the observed as well as CHIRP2 and CHIRP3 standard deviations of 10-day (dekadal) precipitation from February to September during 1981–2019 across 94 Ethiopian stations. The CHIRP2 standard deviations clearly fall to the left of the 1-to-1 line, indicating a substantial underestimation of the precipitation variability. The CHIRP3 standard deviations tend to cluster along the 1-to-1 line. Figure 7b displays the observed and CHIRP2 and CHIRP3 1981–2019 90^th^ percentile precipitation values, at the station locations. CHIRP2 values tend to fall to the left of the 1-to-1 line, while CHIRP3 tends to lie close to this line. These results, along with REGEN-based evaluations (Fig. 4, Tables 7–10) support the superiority of the CHIRP3 algorithm.

### Comparisons of global CHIRPS3, CHIRPS2 and the GPCC Full v2022 Datasets

**Fig. 8:**
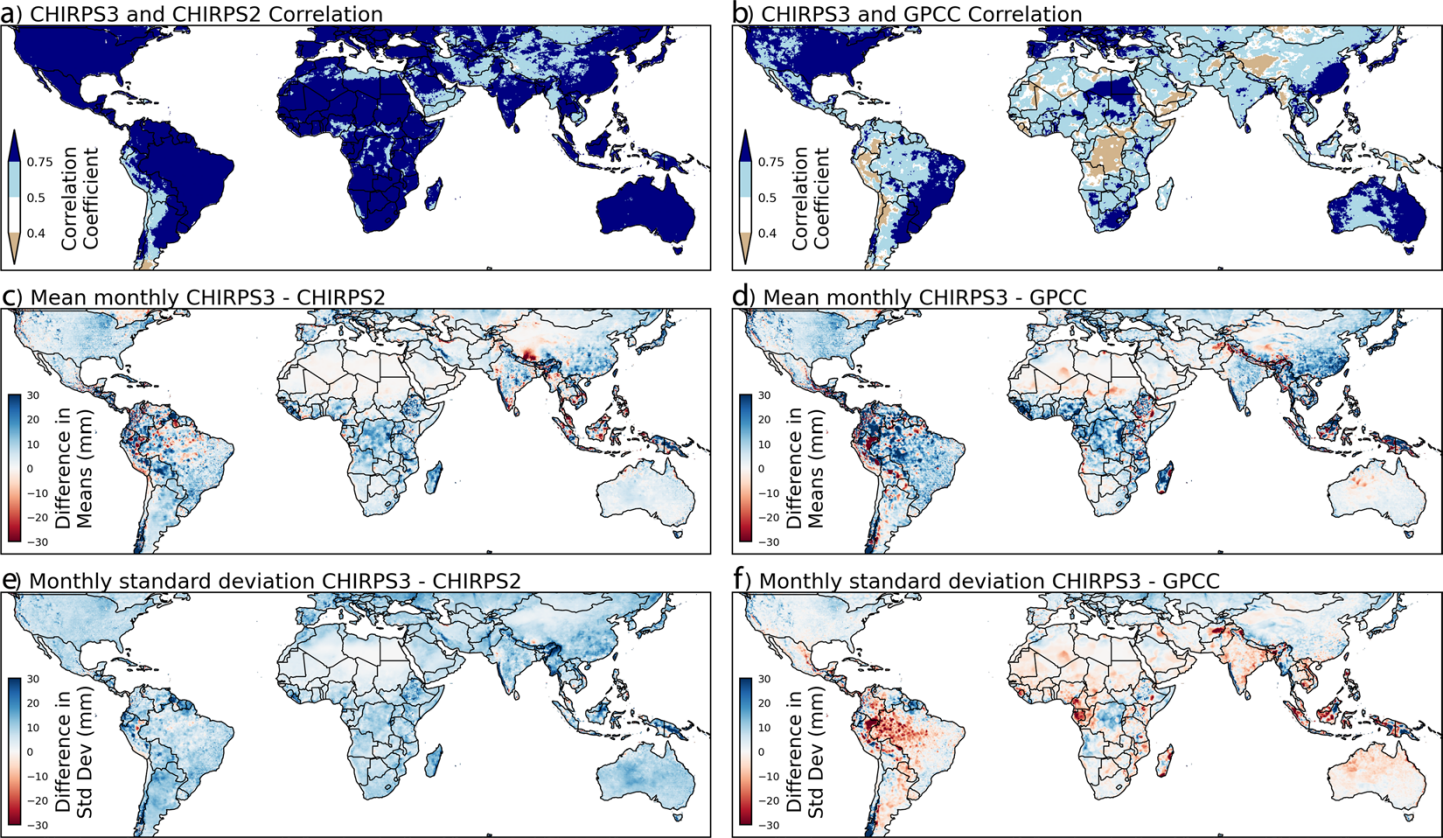
Comparisons of ¼° 1981–2020 monthly CHIRPS3, CHIRPS2 and GPCC Full v2022 data. (**a**) Correlations between monthly CHIRPS3 and CHIRPS2 precipitation anomalies. (**b**) Same, but for CHIRPS3 and GPCC. (**c**) The difference between monthly mean CHIRPS3 and CHIRPS2 (CHIRPS3-CHIRPS2). (**d**) Same, but for CHIRPS3 and GPCC (CHIRPS3-GPCC). (**e**) The difference between the standard deviation of monthly CHIRPS3 and CHIRPS2 anomalies (σ_CHIRPS3_-σ_CHIRPS2_). **f**. Same, but for CHIRPS3 and GPCC (σ_CHIRPS3_-σ_GPCC_).

Figure 8 summarizes a comparison of 1981–2020 CHIRPS3, CHIRPS2 and the GPCC Full v2022 (hereafter GPCC) gridded precipitation archive. The CHIRPS data have been resampled to the GPCC’s ¼° resolution. The results in panels A, B, E and F are based on monthly anomalies, derived using the monthly 1981–2020 mean fields. The correlation between the CHIRPS3 anomalies and CHIRPS2 anomalies (Fig. 8a) is high, with a mean correlation of 0.83. The correlation between the CHIRPS3 and GPCC is also quite high, with an average value of 0.65, but the associated correlation map (Fig. 8b), is lower in poorly gauged regions (Fig. 2).

Figure 8c displays the differences in the mean monthly CHIRPS3 and CHIRPS2 precipitation. Overall, CHIRPS3 is modestly wetter than CHIRPS2, with global mean values of 77.6 mm and 70.9 mm month^−1^. These differences are mainly due to the transition to gauge-undercatch correction in both the CHP_clim_ v2 and the station data blending procedures. Hence, the differences in Fig. 8c tend to arise in windy areas with complex terrain. The GPCC means are, on average, more similar to CHIRPS3 (Fig. 8d), because the GPCC also uses gauge catch under-correction. The GPCC mean precipitation is 77.7 mm month^−1^. Spatially, the largest differences between the GPCC and CHIRPS3 tend to occur in sub-Saharan Africa, Latin America and South America, presumably due to the influence of the mean IMERG precipitation gradients.

Figure 8e displays the differences in the standard deviations of monthly CHIRPS3 and CHIRPS2 precipitation anomalies. Overall, CHIRPS3 is more variable than CHIRPS2, with global mean standard deviations of 35.2 and 27.1 mm month^−1^. Such differences relate both to the higher mean precipitation and the change in the CHIRP3 versus CHIRP2 algorithm. Interestingly, the GPCC standard deviations are, for the most part, lower than CHIRPS3. These areas of lower variability tend to align with areas of lower station density. In these regions, CHIRPS3 will relax towards CHIRP3, whereas GPCC estimates will relax to the background climatology. The GPCC mean standard deviation is 34.8 mm month^−1^.

## Usage Notes

### An example: gauge-undercatch correction for validation studies

In this section we evaluate the performance of CHIRPS2 and CHIRPS3 precipitation products in the Tensift River Basin, Morocco. Using gridded correction fields and independent station data, we compare their ability to capture monthly and annual precipitation patterns. The Tensift River Basin, located in central-west Morocco, spans approximately 26,035 km² and is characterized by a semi-arid to arid climate with Mediterranean influence. Rainfall varies from 503 mm year^−1^ in the High Atlas to 300 mm year^−1^ in the coastal zone, with mean annual temperatures ranging from 17 °C to 20 °C. For this study, monthly rainfall data from 23 well-distributed stations (1982–2022) were selected based on data continuity, quality, and representation of the basin’s diverse topography. Given the limited blending of local stations and the potential impact of gauge-undercatch—particularly in mountainous areas like the High Atlas—a gauge-undercatch correction was necessary to improve CHIRPS3 accuracy before evaluation. To address this, monthly Legates-Willmott gauge-undercatch correction factors^40^ were applied to CHIRPS3. These global half-degree fields are available at: https://data.chc.ucsb.edu/products/CHIRPS/v3.0/diagnostics/legates-willmott_corrections/.

The 12 GeoTIFFs were first merged into a NetCDF file, then clipped to the Tensift Basin, and resampled to match the CHIRPS grid. CHIRPS3 rainfall values were then adjusted by dividing them by the corresponding monthly correction factors, which ranged between 1.00 and 1.50 within the Tensift Basin.

**Fig. 9:**
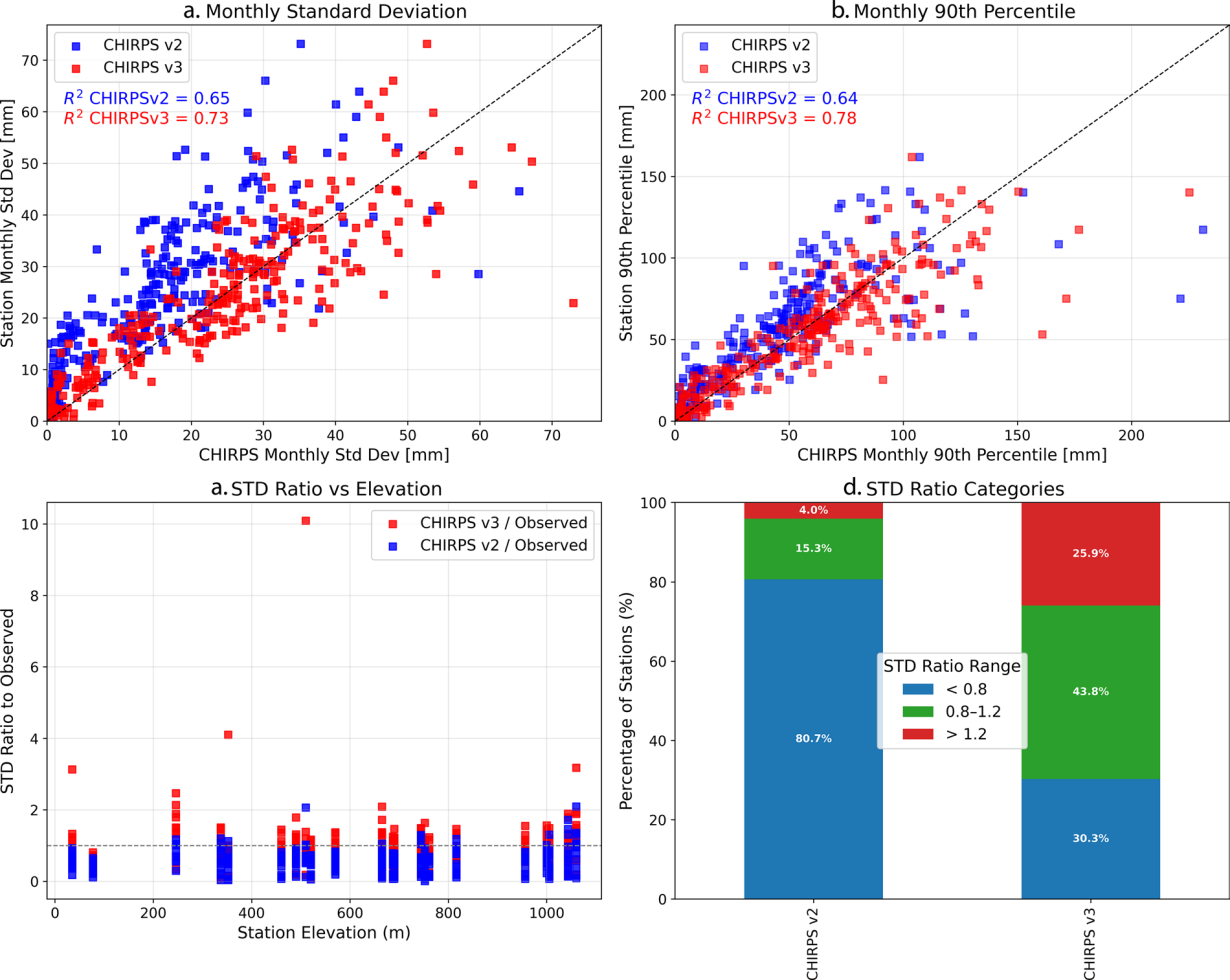
Monthly comparison of CHIRPS3 and CHIRPS2 with observed station data in the Tensift River Basin, Morocco. (**a**) Scatterplot comparing monthly standard deviation (STD) of observed station data with CHIRPS2 and CHIRPS3. (**b**) Same, as (**a**) but comparing the 90th percentile values. (**c**) Standard deviation ratio plotted against station elevation. (**d**) Distribution of stations across three standard deviation ratio categories: below (<0.8), within (0.8–1.2), and above (>1.2) the target range.

In Fig. 9(a–d), we evaluate the agreement between CHIRPS v2 and v3 and station observations in terms of monthly variability and extremes. Panels A and B show strong correlations between CHIRPS estimates and observed monthly standard deviation and 90th percentiles, with CHIRPS3 showing improved performance (R² = 0.73 for STD and 0.78 for P90) compared to CHIRPS2 (R² = 0.65 and 0.64). Panel C shows that CHIRPS2 generally underestimates variability, particularly at stations located above 400 m, while CHIRPS3 provides a more accurate spread of standard deviation across elevations. This is further supported by panel D, where 43.8% of CHIRPS3 values fall within the ideal STD ratio range (0.8–1.2), compared to only 15.3% for CHIRPS2. Moreover, CHIRPS2 has a high proportion (80.7%) of stations below the acceptable variability threshold, highlighting a consistent underestimation of monthly rainfall fluctuations. These results indicate a clear improvement in CHIRPS3’s ability to capture temporal variability and intensity across varied terrain in the Tensift Basin.

**Fig. 10:**
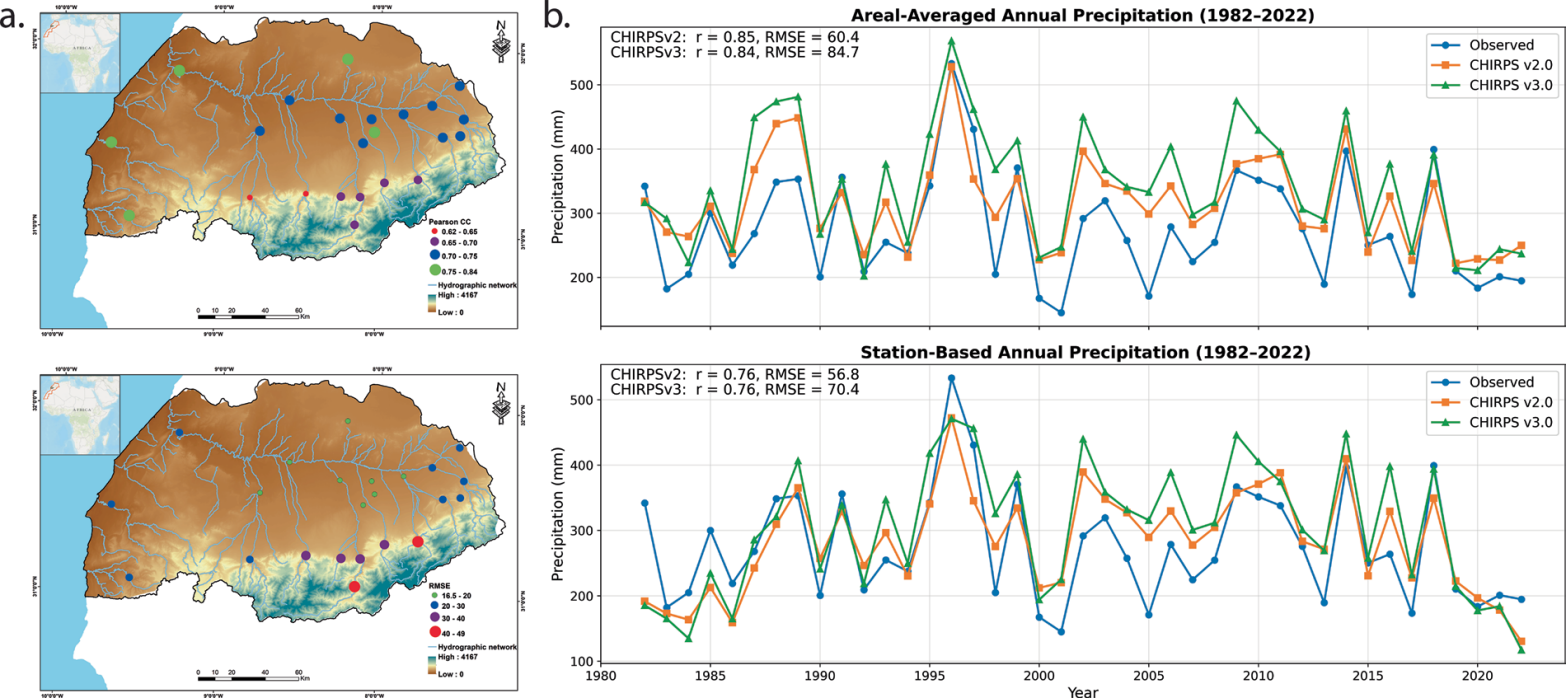
Spatial and temporal comparison of CHIRPS2, CHIRPS3, and observed precipitation in the Tensift River Basin, Morocco. (**a**) Spatial distribution of performance metrics (top panel Pearson correlation and bottom panel RMSE) for CHIRPS v3 based on point-to-pixel comparisons at station locations. (**b**) Comparison of CHIRPS2 and CHIRPS3 annual precipitation with areal-averaged (top panel) and point-based (bottom panel) estimates from the station observations for the period 1982–2022.

In Fig. 10a, the spatial maps highlight station-level performance across the Tensift Basin using CHIRPS3. Pearson correlation and RMSE values indicate that while several stations show moderate to high agreement with observed data (r ranging from 0.62 to 0.84), the performance varies with elevation and topography. Stations located in central and southern mountainous areas (elevation > 1000 m) tend to exhibit higher RMSEs and slightly lower correlation values compared to lower-elevation and flatter regions, suggesting spatial variability in CHIRPS3 performance likely influenced by topographic complexity. It is also important to note that none of the selected stations used in this evaluation are part of the global gauge network incorporated in CHIRPS2 and CHIRPS3 blending (https://data.chc.ucsb.edu/products/). Only three stations from an independent source are included in CHIRPS, and none of them are located in mountainous areas—providing limited ground reference for evaluating CHIRPS accuracy in high-elevation zones.

In Fig. 10b, the temporal plots of annual areal-averaged and point-based precipitation (1982–2022) illustrate the differences in agreement between satellite-based and observed precipitation. For areal-averaged precipitation, CHIRPS2 and CHIRPS3 show similar correlations with observed data (R = 0.85 and 0.84, respectively), though with differing RMSE values (60.4 mm vs. 84.7 mm). A similar pattern holds for point-based estimates, with both versions exhibiting the same correlation (R = 0.76), but RMSE values of 56.8 mm for CHIRPS2 and 70.4 mm for CHIRPS3. These results reflect differences in performance metrics depending on whether station-level or spatially averaged comparisons are used, highlighting the influence of spatial resolution and aggregation methods on validation outcomes.

The CHIRPS2 products are widely used to identify at-risk populations, while supporting anticipatory interventions, agricultural advisories, insurance products, and social safety nets. The CHIRPS3 development team sought to make version 3 better, but not too different from version 2. Using REGEN to clearly identify a systematic problem in version 2 (underestimation of variance) was key to its improvement. Many alternative algorithms were explored, but a very simple approach worked as well, or better, than the alternatives examined. Hence, a simple ratio of mean pentadal precipitation and mean pentadal CCD forms the core of CHIRP3. It is surprising how well such a simple approach performs, capturing very well the REGEN-observed standard deviations and the REGEN-observed 90^th^ percentile values. In Ethiopia, station data indicated similar results.

In addition to increased variance, CHIRPS3 also incorporates many additional stations, at both monthly and sub-monthly time-scales. Also, this new version extends to 60°S–60°N, and uses gauge-undercatch corrected station data.

When validating the CHIRPS3 products, or transitioning from CHIRPS2 to CHIRPS3, it is important to remember that CHIRPS3 will tend to be systematically wetter than CHIRPS2, by construction, because both the CHP_clim_ v2 climatology and the blended station data are gauge-undercatch corrected. Section 5.1 provided an example of gauge-undercatch correction grids can be used to assess the accuracy of CHIRPS3.

Like CHIRPS2, CHIRPS3 ‘stacks’ information from a high-resolution satellite-enhanced climatology, TIR precipitation estimates, and gauge observations. Each of these components have been improved in version 3. The CHIRPS3 product benefits from many station data contributors (Table 1), the Climate Hazard Center validation team, the GridSat B1^31^ and CPC^32^ TIR data, ERA5^42^, and the training information contained in the IMERG dataset^39^. The CHIRPS products have been designed for the public good, supported by a broad set of contributors (Table 1, Table 3)^48–54^.

## Data Availability

The Climate Hazards Center Infrared Precipitation with Stations (CHIRPS v3) is a 40 + year, high-resolution quasi-global rainfall dataset. It spans 60°N to 60°S and covers all longitudes, providing data from 1981 to near-present. CHIRPS v3 combines satellite-based thermal infrared rainfall estimates with *in-situ* station observations to produce a 0.05° gridded rainfall time series over land. Two CHIRPS products are available – a rapidly updated preliminary product and a final product. CHIRPS Preliminary incorporates rapidly updated station observations and is available 2 days after the end of a pentad (on the 2nd, 7th, 12th, 17th, 22nd and 27th day of each month). The CHIRPS v3 final product blends in best available station inputs and is produced once a month, typically on the third week of the following month. The dataset covers the global domain, with additional sub-domain products available for Africa and Latin America. CHIRPS v3 is available in several formats (GeoTIFF, NetCDF, BIL, and COG) and multiple timesteps (daily, pentad, dekad, monthly and annual). CHIRPS is fundamentally a pentad and monthly product, and all other time-steps are derived from those. Based on two different ways of downscaling CHIRPS v3, we provide two different daily products: A reanalysis ‘rnl’ and a satellite ‘sat’ product. The ‘rnl’ uses daily precipitation from the ECMWF ERA Reanalysis v5 (ERA5) data product to partition pentadal CHIRPS-v3 precipitation totals into daily amounts. The ‘sat’ uses daily precipitation from the NASA IMERG Late V07 data product (IMERG) to partition pentadal CHIRPS-v3 precipitation totals into daily amounts. More information can be found at https://data.chc.ucsb.edu/products/CHIRPS/v3.0/daily/readme.txt. These data can be found at the UCSB CHC CHIRPS3 webpage (https://www.chc.ucsb.edu/data/chirps3), where the CHIRPS3 data repository is also located. The CHIRPS 3 has the following Digital Object Identifier: 10.15780/G2JQ0P^43^ CHIRPS3 is in the public domain, as registered with Creative Commons, and is under a Creative Commons Attribution 4.0 International License. To the extent possible under the law, the Climate Hazards Center has waived all copyright and related or neighboring rights to CHIRPS3.

## Code availability

The code used to produce the CHIRPS3 product has been written the ‘Interactive Data Langauge’ (IDL). IDL is a commercial scientific programming language, supported by NV5 Geospatial Software (https://www.nv5geospatialsoftware.com/Products/IDL). The CHIRPS3 IDL source code is available at https://data.chc.ucsb.edu/products/CHIRPS/v3.0/diagnostics/chirps-v3.code.tar. Note that the CHIRPS blending and interpolation process, as represented by this code, is not intended to be an installable package.

## References

[1] Ross, K., Brown, M. E., Verdin, J. P. & Underwood, L. Review of FEWS NET biophysical monitoring requirements. *Environ Res Lett* **4**, 024009 (2009).

[2] Verdin, J., Funk, C., Senay, G. & Choularton, R. Climate science and famine early warning. *Philos T Roy Soc B* **360**, 2155–2168 (2005). 10.1098/rstb.2005.1754 PMC1569579 16433101

[3] Funk, C. *et al*. A global satellite assisted precipitation climatology. *Earth Syst. Sci. Data Discuss.* **7**, 1–13, 10.5194/essdd-7-1-2015 (2015).

[4] Funk, C. *et al*. A Quasi-global Precipitation Time Series for Drought Monitoring. (2014).

[5] Funk, C. *et al*. The climate hazards infrared precipitation with stations—a new environmental record for monitoring extremes. *Scientific data* **2** (2015). 10.1038/sdata.2015.66 PMC4672685 26646728

[6] Huffman, G. J. *et al*. Integrated multi-satellite retrievals for the global precipitation measurement (GPM) mission (IMERG). *Satellite precipitation measurement: Volume 1*, 343–353 (2020).

[7] Hong, Y., Hsu, K.-L., Sorooshian, S. & Gao, X. Precipitation Estimation from Remotely Sensed Imagery Using an Artificial Neural Network Cloud Classification System. *Journal of Applied Meteorology* **43**, 1834–1853, 10.1175/JAM2173.1 (2004).

[8] Beck, H. E. *et al*. MSWEP V2 global 3-hourly 0.1 precipitation: methodology and quantitative assessment. *B Am Meteorol Soc* **100**, 473–500 (2019).

[9] Becker, A. *et al*. A description of the global land-surface precipitation data products of the Global Precipitation Climatology Centre with sample applications including centennial (trend) analysis from 1901-present. *Earth System Science Data* **5**, 71–99 (2013).

[10] Huffman, G. J. *et al*. The new version 3.2 global precipitation climatology project (GPCP) monthly and daily precipitation products. *Journal of Climate* **36**, 7635–7655 (2023).

[11] Harrison, L. *et al*. Advancing early warning capabilities with CHIRPS-compatible NCEP GEFS precipitation forecasts. *Scientific Data* **9**, 1–13 (2022). 35773449 10.1038/s41597-022-01468-2 PMC9246965

[12] Shukla, S. *et al*. Enhancing the application of earth observations for improved environmental decision-making using the Early Warning eXplorer (EWX). *Frontiers in Climate* **2**, 583509 (2021).

[13] Agutu, N. *et al*. Assessing multi-satellite remote sensing, reanalysis, and land surface models’ products in characterizing agricultural drought in East Africa. *Remote sensing of environment* **194**, 287–302 (2017).

[14] Beck, H. E. *et al*. Daily evaluation of 26 precipitation datasets using Stage-IV gauge-radar data for the CONUS. *Hydrology and Earth System Sciences* **23**, 207–224 (2019).

[15] Beck, H. E. *et al*. Global-scale evaluation of 22 precipitation datasets using gauge observations and hydrological modeling. *Hydrology and Earth System Sciences* **21**, 6201 (2017).

[16] Dinku, T. *et al*. Validation of the CHIRPS satellite rainfall estimates over eastern of Africa. *Quarterly Journal of the Royal Meteorological Society* (2018).

[17] Duan, Z., Liu, J., Tuo, Y., Chiogna, G. & Disse, M. Evaluation of eight high spatial resolution gridded precipitation products in Adige Basin (Italy) at multiple temporal and spatial scales. *Science of the Total Environment* **573**, 1536–1553 (2016). 27616713 10.1016/j.scitotenv.2016.08.213

[18] Duan, Z. *et al*. Hydrological evaluation of open-access precipitation and air temperature datasets using SWAT in a poorly gauged basin in Ethiopia. *Journal of hydrology* **569**, 612–626 (2019).

[19] Gao, F. *et al*. Comparison of two long-term and high-resolution satellite precipitation datasets in Xinjiang, China. *Atmospheric Research* **212**, 150–157 (2018).

[20] Gummadi, S., Dinku, T., Shirsath, P. B. & Kadiyala, D. M. Spatial and Temporal Evaluation of Satellite Rainfall Estimates Over Vietnam. (2021).

[21] Harrison, L., Funk, C. & Peterson, P. Identifying changing precipitation extremes in Sub-Saharan Africa with gauge and satellite products. *Environ Res Lett* **14**, 085007 (2019).

[22] Paredes Trejo, F. J., Alves Barbosa, H., Peñaloza-Murillo, M. A., Moreno, M. A. & Farias, A. Intercomparison of improved satellite rainfall estimation with CHIRPS gridded product and rain gauge data over Venezuela. *Atmósfera* **29**, 323–342 (2016).

[23] Prakash, S. Performance assessment of CHIRPS, MSWEP, SM2RAIN-CCI, and TMPA precipitation products across India. *Journal of hydrology* **571**, 50–59 (2019).

[24] Retalis, A., Katsanos, D., Tymvios, F. & Michaelides, S. Validation of the first years of GPM operation over Cyprus. *Remote Sensing* **10**, 1520 (2018).

[25] Rivera, J. A., Marianetti, G. & Hinrichs, S. Validation of CHIRPS precipitation dataset along the Central Andes of Argentina. *Atmospheric Research* **213**, 437–449 (2018).

[26] Shrestha, N. K. *et al*. Evaluating the accuracy of Climate Hazard Group (CHG) satellite rainfall estimates for precipitation based drought monitoring in Koshi basin, Nepal. *Journal of Hydrology: Regional Studies* **13**, 138–151 (2017).

[27] Funk, C. C. *et al*. Introducing and Evaluating the Climate Hazards Center IMERG with Stations (CHIMES): Timely Station-Enhanced Integrated Multisatellite Retrievals for Global Precipitation Measurement. *B Am Meteorol Soc* **103**, E429–E454, 10.1175/BAMS-D-20-0245.1 (2022).

[28] Fuchs, T., Rapp, J., Rubel, F. & Rudolf, B. Correction of synoptic precipitation observations due to systematic measuring errors with special regard to precipitation phases. *Physics and Chemistry of the Earth, Part B: Hydrology, Oceans and Atmosphere* **26**, 689–693 (2001).

[29] Maidment, R. I., Allan, R. P. & Black, E. Recent observed and simulated changes in precipitation over Africa. *Geophysical Research Letters* **42**, 8155–8164 (2015).

[30] Maidment, R. I. *et al*. The 30 year TAMSAT African Rainfall Climatology And Time series (TARCAT) data set. *Journal of Geophysical Research: Atmospheres* **119**, 10,619–610,644 (2014).

[31] Knapp, K. R. *et al*. Globally gridded satellite (GriSat) observations for climate studies. *Bulletin of the American Meteorological Society* **92**, 893–907 (2011).

[32] Janowiak, J. E., Joyce, R. J. & Yarosh, Y. A real-time global half-hourly pixel-resolution infrared dataset and its applications. *Bull. Amer. Meteor. Soc.* **82**, 205–217 (2001).

[33] Huffman, G. J. *et al*. The TRMM Multisatellite Precipitation Analysis (TMPA): Quasi-Global, Multiyear, Combined-Sensor Precipitation Estimates at Fine Scales. *Journal of Hydrometeorology* **8**, 38–55 (2007).

[34] Menne, M. J., Williams, C. N., Gleason, B. E., Rennie, J. J. & Lawrimore, J. H. The global historical climatology network monthly temperature dataset, version 4. *Journal of Climate* **31**, 9835–9854 (2018).

[35] Menne, M. J., Durre, I., Vose, R. S., Gleason, B. E. & Houston, T. G. An overview of the global historical climatology network-daily database. *Journal of Atmospheric and Oceanic Technology* **29**, 897–910 (2012).

[36] Applequist, S., Durre, I. & Vose, R. The Global Historical Climatology Network Monthly Precipitation Dataset, Version 4. *Scientific Data* **11**, 633 (2024). 38879587 10.1038/s41597-024-03457-z PMC11180175

[37] Schneider, U. *et al*. GPCC’s new land surface precipitation climatology based on quality-controlled *in situ* data and its role in quantifying the global water cycle. *Theoretical and Applied Climatology* **115**, 15–40 (2013).

[38] Schneider, U. *et al*. Evaluating the Hydrological Cycle over Land Using the Newly-Corrected Precipitation Climatology from the Global Precipitation Climatology Centre (GPCC). *Atmosphere* **8**, 52 (2017).

[39] Huffman, G. J. *et al*. NASA global precipitation measurement (GPM) integrated multi-satellite retrievals for GPM (IMERG). *Algorithm theoretical basis document, version* **4**, 30 (2015).

[40] Legates, D. R. & Willmott, C. J. Mean seasonal and spatial variability in gauge‐corrected, global precipitation. *International Journal of Climatology* **10**, 111–127 (1990).

[41] Arkin, P. A. & Meisner, B. N. The relationship between large-scale convective rainfall and cold cloud over the western hemisphere during 1982-84. *Monthly Weather Review* **115**, 51–74 (1987).

[42] Hersbach, H. *et al*. The ERA5 global reanalysis. *Quarterly Journal of the Royal Meteorological Society* **146**, 1999–2049 (2020).

[43] Funk, C. *et al*. CHIRPS: Rainfall Estimates from Rain Gauge and Satellite Observations. *Climate Hazards Center*. 10.15780/G2JQ0P (2025).

[44] Contractor, S. *et al*. Rainfall Estimates on a Gridded Network (REGEN)–a global land-based gridded dataset of daily precipitation from 1950 to 2016. *Hydrology and Earth System Sciences* **24**, 919–943 (2020).

[45] Roca, R. *et al*. FROGS: a daily 1° × 1° gridded precipitation database of rain gauge, satellite and reanalysis products. *Earth Syst. Sci. Data* **11**, 1017–1035, 10.5194/essd-11-1017-2019 (2019).

[46] Dinku, T. *et al*. Validation of the CHIRPS satellite rainfall estimates over eastern Africa. *Quarterly Journal of the Royal Meteorological Society* **144**, 292–312 (2018).

[47] Toté, C. *et al*. Evaluation of satellite rainfall estimates for drought and flood monitoring in Mozambique. *Remote Sensing* **7**, 1758–1776 (2015).

[48] Funk, C. *et al*. Examining the role of unusually warm Indo‐Pacific sea‐surface temperatures in recent African droughts. *Quarterly Journal of the Royal Meteorological Society* **144**, 360–383 (2018).

[49] Nicholson, S. E., Fink, A. H., Funk, C., Klotter, D. A. & Satheesh, A. R. Meteorological causes of the catastrophic rains of October/November 2019 in equatorial Africa. *Global and Planetary Change* **208**, 103687 (2022).

[50] Husak, G. J., Funk, C. C., Michaelsen, J., Magadzire, T. & Goldsberry, K. P. Developing seasonal rainfall scenarios for food security early warning. *Theoretical and Applied Climatology*, 1–12 (2013).

[51] Senay, G. B. & Verdin, J. Characterization of yield reduction in Ethiopia using a GIS-based crop water balance model. *Canadian Journal of Remote Sensing* **29**, 687–692 (2003).

[52] McNally, A. *et al*. A land data assimilation system for sub-Saharan Africa food and water security applications. *Scientific data* **4**, 170012 (2017). 28195575 10.1038/sdata.2017.12 PMC5308203

[53] Verdin, A. *et al*. Development and validation of the CHIRTS-daily quasi-global high-resolution daily temperature data set. *Scientific Data* **7**, 303 (2020). 32929097 10.1038/s41597-020-00643-7 PMC7490712

[54] Funk, C. *et al*. A high-resolution 1983–2016 T max climate data record based on infrared temperatures and stations by the Climate Hazard Center. *Journal of Climate* **32**, 5639–5658 (2019).

[55] Lawrimore, J. H. *et al*. An overview of the Global Historical Climatology Network monthly mean temperature data set, version 3. *Journal of Geophysical Research: Atmospheres* **116** (2011).

[56] GSOD. *Global Surface Summary of the Day (GSOD), Version 1.0.* https://www.ncei.noaa.gov/access/metadata/landing-page/bin/iso?id=gov.noaa.ncdc:C00516 (2025).

[57] Menne, M. J. *et al*. *Global historical climatology network-daily (GHCN-Daily), Version 3*, (2012).

[58] CPC. *‘NOAA Climate Prediction Center, Climate Anomaly Database Version 2 (CADB_v2) Global Station Observation Summaries*., https://www.cpc.ncep.noaa.gov/products/cadb/ (2025).

[59] SWALIM. *Somalia Land and Water Information System (SWALIM)*, https://faoswalim.org/ (2025).

[60] Conagua. *Comisión Nacional del Agua*, https://www.gob.mx/conagua (2025).

[61] EMI. *Ethiopian Meteorological Institute*, https://www.ethiomet.gov.et/ (2025).

[62] ChileMet. *Dirección Meteorológica de Chile*, https://www.meteochile.gob.cl/PortalDMC-web/index.xhtml (2025).

[63] IMHPA. *Instituto de Meteorología e Hidrología de Panamá*, https://imhpasig.maps.arcgis.com/home/index.html (2025).

[64] INSIVUMEH. *Instituto Nacional de Sismología, Vulcanologia, Meteorologia e Hidrología*, https://insivumeh.gob.gt/ (2025).

[65] IDEAM. *Instituto de Hidrología, Meteorología y Estudios Ambientales*, (2025).

[66] MCDW. *Monthly Climatic Data for the World (MCDW)*., https://www.ncei.noaa.gov/access/metadata/landing-page/bin/iso?id=gov.noaa.ncdc:C01037 (2025).

[67] ZMSD. *Meteorological Services Department of Zimbabwe* (2025).

[68] KMD. *Kenya Meteorological Department, 3D Printed Automatic Weather Stations (3D PAWS)*, https://3d-fewsnet.icdp.ucar.edu/dashboard (2025).

[69] CEMADEN. *Centro Nacional de Monitoramento e Alertas de Desastres Naturais*, https://www.gov.br/cemaden/pt-br (2025).

[70] COPECO. *Comité Permanente de Contigencias*, https://copeco.gob.hn/ (2025).

[71] IMN. *Instituto Meteorológico Nacional de Costa Rica*, https://www.imn.ac.cr/web/imn/inicio (2025).

[72] INAM. *Instituto Nacional de Meteorologia*, https://www.inam.gov.mz/index.php/pt/ (2025).

[73] INMET. *Instituto Nacional de Meteorologia*, https://portal.inmet.gov.br/ (2025).

[74] KMA. *Korea Meteorological Administration*, https://www.wamis.go.kr/ (2025).

[75] SISSA. *System for southern South America*, https://sissa.crc-sas.org/ (2025).

[76] NMS. *National Meteorological Service of Belize*, https://nms.gov.bz/ (2025).

[77] NMA. *National Meteorological Agency - Somalia*, https://meteosomalia.so/ (2025).

[78] CanadaMet. *Meteorological Service of Canada*, https://climate.weather.gc.ca/prods_servs/cdn_climate_summary_e.html (2025).

[79] (SEPA), S. E. P. A. *Scottish Environment Protection Agency*, https://www.sepa.org.uk/ (2025).

[80] DWD. *Germany National Meteorological Service*, https://opendata.dwd.de/climate_environment/CDC/observations_germany/climate/daily/more_precip/ (2025).

